# Transcriptional profiling of molecular pathways allows for the definition of robust lung squamous cell carcinoma molecular subtypes with specific vulnerabilities

**DOI:** 10.1002/ctm2.1413

**Published:** 2023-09-21

**Authors:** Sara Hijazo‐Pechero, Ania Alay, David Cordero, Raúl Marín, Noelia Vilariño, Ramón Palmero, Jesús Brenes, Ernest Nadal, Xavier Solé

**Affiliations:** ^1^ Unit of Bioinformatics for Precision Oncology Catalan Institute of Oncology (ICO) L'Hospitalet de Llobregat Spain; ^2^ Preclinical and Experimental Research in Thoracic Tumors (PrETT), Molecular Mechanisms and Experimental Therapy in Oncology Program (Oncobell) Bellvitge Biomedical Research Institute (IDIBELL) L'Hospitalet de Llobregat Spain; ^3^ Translational Genomics and Targeted Therapies in Solid Tumors August Pi i Sunyer Biomedical Research Institute (IDIBAPS) Barcelona Spain; ^4^ Thoracic Oncology Unit Catalan Institute of Oncology (ICO) Barcelona Spain; ^5^ Neuro‐Oncology Unit Catalan Institute of Oncology (ICO) L'Hospitalet de Llobregat Spain; ^6^ Molecular Biology CORE, Center for Biomedical Diagnostics (CDB) Hospital Clínic de Barcelona Barcelona Spain

Dear Editor,

Lung squamous cell carcinoma (SCC) is a histological subtype of non‐small cell lung cancer associated with poor prognosis. Actionable driver alterations are extremely rare in SCC and standard of care (SoC) consists of immunotherapy alone or combined with chemotherapy based on the PD‐L1 expression, with few long‐term survivors. We hypothesized that transcriptomic data analysis can improve patient stratification and may unravel novel treatment approaches for these patients.^1^

We developed a bioinformatics pathway‐based classification framework using publicly available whole‐transcriptome data from more than 2,000 SCC samples focusing on 50 pathways. Previous transcriptome‐based classifications used individual gene expression measures, which are prone to multiple sources of variability.^2^ Detailed methodology, gene expression datasets and schematic view of the bioinformatics framework are shown in Table S1 and Figures S1 and S2, respectively. Five SCC subtypes were identified based on the combined transcriptional behaviour of the 50 pathways (Figure 1A,B): SCC1 (9.9% of patients), SCC2 (23.9%), SCC3 (25.8%), SCC4 (31.0%) and SCC5 (9.4%). SCC subtypes displayed their specific transcriptional footprint, which could shape different treatment responses (Figure 1C and Figure S3). SCC1 and SCC4 showed higher activation levels of cell proliferation and DNA damage response (DDR) pathways, and rather low transcriptional activation levels of immune‐related programs, especially SCC4. In contrast, SCC2 showed higher immune system‐related pathways activation and lower cell cycle signatures activation. SCC3 exhibited high activity of proliferation and immune‐related pathways, and upregulation of KRAS, NFKB, IL2‐STAT5 and TNFA signaling pathways, which may play a role in shaping these tumours' immunity. SCC5 displayed reduced activation of most pathways. Our classification partly overlaps with previous intrinsic subtypes described by Wilkerson et al,^3^ where primitive subtype correlates with the proliferative SCC4, while secretory subtype was distributed between the immune‐enriched SCC2 and SCC3, and the classical subtype overlapped with SCC3 and SCC4 (Figure S4). No significant differences were observed in clinicopathological characteristics or overall survival (Table S2 and Figure S5). This underlines the importance of this work aiming to distinguish consistent groups within an apparently clinically homogeneous population of patients with SCC and unravel potential transcriptional vulnerabilities.

**FIGURE 1:**
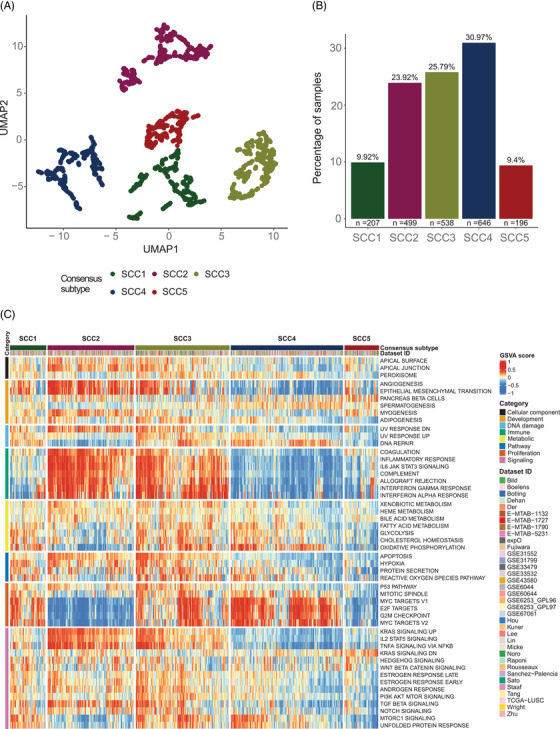
Overview of squamous cell carcinoma (SCC) groups at the transcriptional level. (A) Final consensus map of lung SCC tumours. Each dot represents the summary centroid of the different subpopulations identified during the classification process. Using UMAP and walktrap clustering method with Euclidean distance on these centroids, five different consensus subtypes represented by different colours were identified based on the joint behaviour of the 50 studied molecular pathways. (B) Distribution of the five identified lung SCC subtypes. (C) Relative activity levels of the 50 studied pathways in each of the 2086 SCC samples were assigned to a consensus subtype. Red colours indicate higher relative activity of a pathway in a certain sample, whereas blue colours indicate lower relative activity of a pathway in a certain sample.

SCC subtypes were further characterized to identify differential genomic patterns (Figure 2). For mutational signatures, tobacco‐related genomic signature was found to be overrepresented across all subtypes, suggesting an equivalent tobacco‐related DNA damage (Figure S6). Although subtle differences were observed regarding tumour mutational burden (TMB), we found higher copy number alterations (CNA) in SCC1 and SCC4 (Figure 2A,B and Tables S3 and S4). SCC1 further demonstrated higher DDR deficiency scores compared to other subtypes (Figure 2C and Table S5). Thus, the greater genomic instability found for SCC1 and SCC4 might not be the result of higher exposure to exogenous carcinogens (i.e. tobacco), but rather a consequence of DDR mechanisms, or replication stress.^4^ This classification framework and the reported association of the subtypes with genomic instability (i.e. higher CNA rates) was validated in an independent dataset of SCC (Figure 3).^5^ All five subtypes were found in the CPTAC‐3 dataset and samples map within one of the consensus subtypes, which supports the robustness and reproducibility of this classification (Figure 3A). We observed that both CNA and pathways activation were concordant in both discovery and validation sets, therefore genomic alterations were consistent beyond the expression patterns used to classify these samples (Figure 3B,C).

**FIGURE 2:**
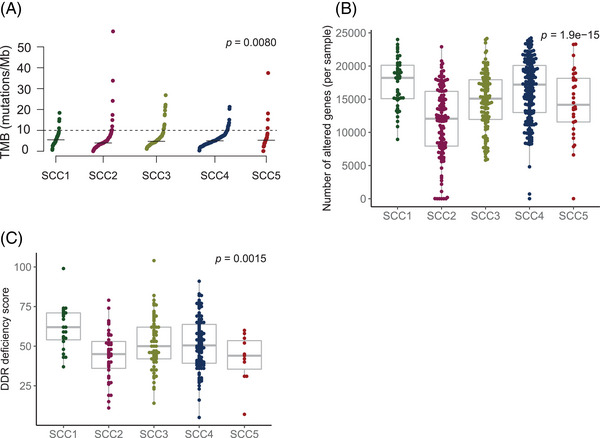
Genomic characterization. (A) Tumor mutational burden (TMB) across lung squamous cell carcinoma (SCC) consensus subtypes. Each dot represents the TMB value for a specific sample. The black segment represents the median TMB value for each lung SCC subtype. Kruskal‐Wallis tests were used to make comparisons between groups. p‐Value was corrected using the false discovery rate (FDR) multiple‐comparisons correction method. (B) Copy number burden across lung SCC subtypes. Each dot represents the number of altered genes per sample. Kruskal‐Wallis tests were used to make comparisons between groups. p‐Value was corrected using the false discovery rate (FDR) multiple‐comparisons correction method. (C) DNA damage repair (DDR) deficiency score distribution across lung SCC subgroups. Each dot represents the DDR score per sample. Kruskal‐Wallis tests were used to make comparisons between groups. p‐Value was corrected using the false discovery rate (FDR) multiple‐comparisons correction method.

**FIGURE 3:**
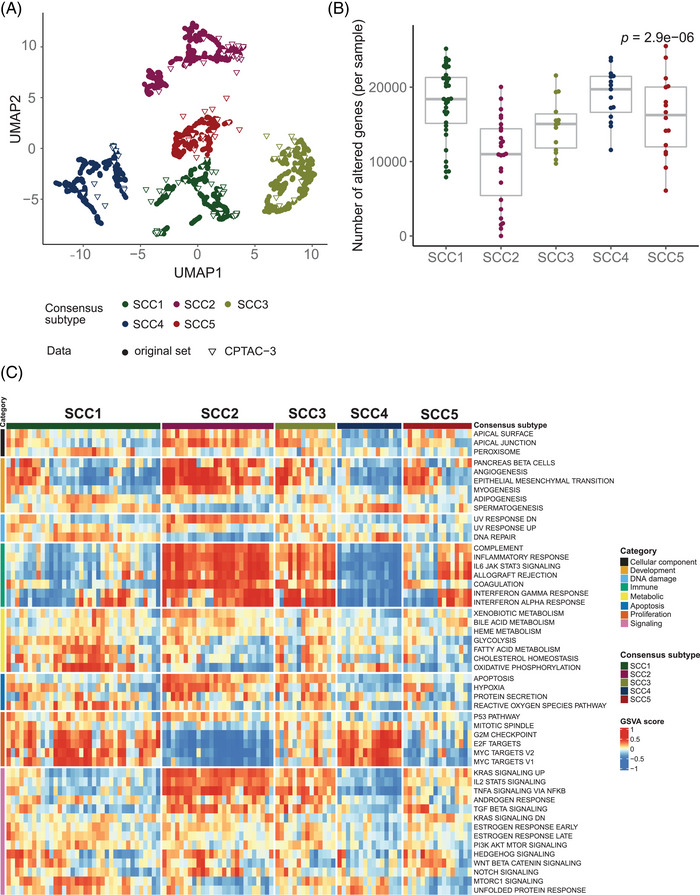
Lung squamous cell carcinoma (SCC) consensus subtypes independent validation. (A) New CPTAC‐3 lung SCC samples were mapped on the previously established classification of SCC tumours based on the activity levels of the same 50 pathways used to define the original SCC subtypes. The new sample subtype status was decided based on the most frequent label of the closest neighbours of the original classification. Coloured circles represent samples used in the original set, whereas triangles represent new CPTAC‐3 validation set samples. (B) Copy number burden across newly classified CPTAC‐3 lung SCC samples. Each dot represents the number of altered genes per sample. The Kruskal‐Wallis test was used to make comparisons between groups. *p*‐Value was corrected using the false discovery rate (FDR) multiple‐comparisons correction method. (C) Relative activity levels of the 50 studied pathways in each of the 108 CPTAC‐3 lung SCC samples were assigned to a consensus subtype. Red colours indicate higher relative activity of a pathway in a certain sample, whereas blue colours indicate lower relative activity of a pathway in a certain sample.

Immune checkpoint inhibitors (ICI) alone, combined with chemotherapy, or following chemoradiotherapy are part of the SoC for advanced SCC.^6^ However, patient selection strategies, based on single biomarkers (i.e. TMB and PD‐L1), fail to predict long‐term clinical benefit in SCC.^7^ We evaluated immune‐cell‐specific signatures and immune‐related gene expression, which revealed different immune landscapes for the subtypes, with potential clinical implications (Figure 4). For instance, SCC2 and SCC3 demonstrated higher infiltration for both anti‐tumour (i.e., cytotoxic, CD8+ and T‐helper 1 cells), and immunosuppressive populations (i.e. M2‐macrophages and T‐regulatory cells), which could eventually prevent an effective immune response (Figure 4A and Figure S7). SCC2 and SCC3 also comprised tumours with high expression of most ICI, including *CD274* (Figure 4B and Figure S8). Although further validation (i.e. scRNA‐Seq) would be needed, these results highlight the need to characterize the immune contexture, along with conventional single biomarkers, to stratify patients and deliver tailored and effective treatment strategies for advanced SCC.

**FIGURE 4:**
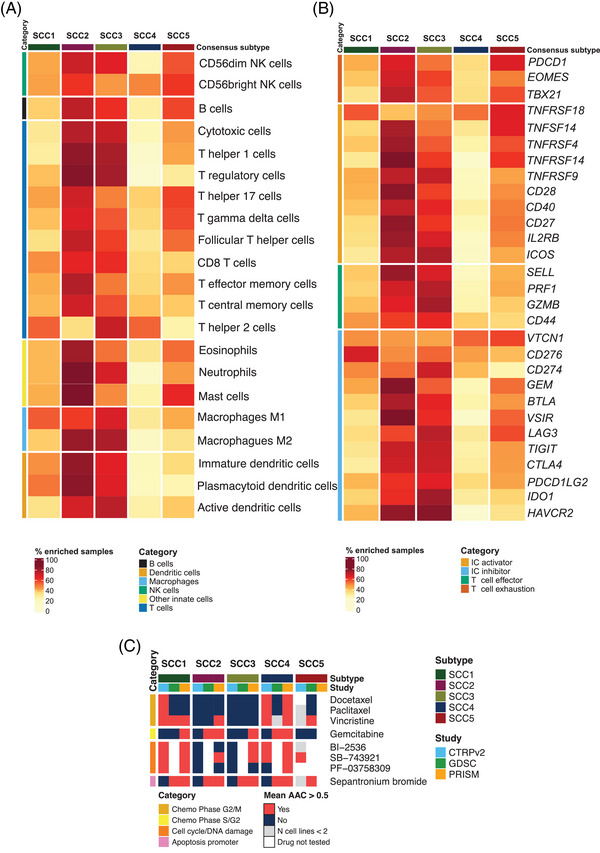
Immune contexture characterization and potential subtype‐specific vulnerabilities. (A) Percentage of samples with high infiltration of each of the 21 evaluated immune cell types. Median immune cell abundance GSVA score values were used as a cut‐off to designate if a sample is enriched for a specific immune cell. Different immune cell categories are represented with different colours on the left side of the heatmap. (B) Percentage of samples with high expression of each of the evaluated immune‐related biomarkers. Median gene expression values for each gene in each gene expression dataset were used as a cut‐off to designate if a sample is enriched for a specific biomarker. Different immune marker categories are represented with different colours on the left side of the heatmap. (C) Potential therapeutic vulnerabilities for the lung squamous cell carcinoma (SCC) subtypes based on CTRPv2, GDSC and PRISM lung SCC cell lines drug sensitivity data. Heatmap representing drugs with mean AAC values greater than 0.5 in at least two studies within the same subtype. Mean AAC values were only calculated if the drug had been tested in at least two different SCC‐CCLs within a subtype and study. Subtypes were considered potentially sensitive to the treatment if the average AAC value for the cell lines classified within a certain group was greater than 0.5 for at least two out of the three evaluated pharmacogenomics studies.

Chemotherapy is also a key treatment for patients with SCC. Understanding chemotherapy response patterns and improving patients’ selection remains crucial. Integrative analysis of pharmacogenomic data in SCC cell lines (SCC‐CCL), showed that the SCC4 subtype might benefit from different chemotherapy regimens (i.e. average AAC above 0.5 in at least two studies), which correlates with the proliferative nature and higher genome instability observed for this subtype (Figure 4C). In this set of SCC cell lines, platinum‐based agents showed AAC values below < 0.2, regardless of the SCC subtype, suggesting lower sensitivity to these compounds (data not shown). However, the evaluation of gene‐expression signatures predicting platinum resistance showed that primary tumors classified as SCC4 and SCC5 would potentially be more sensitive to these chemotherapies (Figure S9).^8^ Moreover, SCC4 and SCC1 SCC‐CCL showed potential sensitivity for some cell cycle and DNA damage‐targeted therapies (Figure 4C).

Single biomarkers and individual gene signatures have shown a limited ability to capture tumour heterogeneity. No pathway was exclusively expressed in one of the subtypes, but their combined expression pattern allowed the identification of subtypes with unique transcriptional footprints. To simplify the classification framework, we derived gene expression signatures for each subtype in the discovery cohort and validated them in the validation cohort (Figure S10 and Table S6). However, these signatures are still complex, and their discrimination ability needs to be further evaluated. We would rather embrace a whole transcriptome technology, applicable at the clinical level (i.e. HTG Edge‐Seq), that enables the evaluation of the combined activity levels of the proposed pathways.

In conclusion, we have presented a comprehensive molecular classification of SCC, based on the transcriptional activity of 50 pathways. Although further validation is required, these results could be useful for improving precision medicine for patients with lung SCC, who have limited treatment options and heterogeneous responses to standard treatments.

## CONFLICT OF INTEREST STATEMENT

Xavier Solé participated in lectures from Roche. Ernest Nadal received research support from Roche, Pfizer, Merck‐Serono and Bristol Myers Squibb; and participated in advisory boards or lectures from Bristol Myers Squibb, Merck Serono, Merck Sharpe & Dohme, Lilly, Roche, Pfizer, Bayer, Sanofi, Takeda, Boehringer Ingelheim, Janssen, Daiichi Sankyo, Amgen and AstraZeneca. The rest of the authors declare no conflict of interest.

## FUNDING INFORMATION

Sara Hijazo‐Pechero is supported by an AGAUR‐FI fellowship (2022 FI_B2 00066), with the support of the FI program of the Secretariat for Universities and Research of the Department of Business and Knowledge of the Government of Catalonia, and the support of the European Union through the European Social Fund “ESF, Investing in your future”. Xavier Solé received support from Ministerio de Ciencia, Innovación y Universidades, which is part of Agencia Estatal de Investigación (AEI), through Retos Research Grant, number RTI2018‐102134‐A‐I00. (Co‐funded by the European Regional Development Fund. ERDF, a way to build Europe). Ernest Nadal received support from Instituto de Salud Carlos III (grants PI18/00920 and PI21/00789) (co‐funded by the European Regional Development Fund. ERDF, a way to build Europe). We thank the CERCA Programme / Generalitat de Catalunya for institutional support. Raúl Marín is supported with the funding of the Ministerio de Universidades, through the predoctoral fellowship number FPU19/01734 for the Formación de Profesorado Universitario (FPU). Noelia Vilariño and Jesús Brenes are supported by a Rio Hortega contract (CM19/00245 & CM21/00073, respectively) from the Instituto de Salud Carlos III.

## Supporting information

Supporting Information

Supporting Information

Supporting Information

## Data Availability

Gene expression data for SCC consensus classification were obtained from GEO and ArrayExpress public repositories. Specific information and identifiers of each dataset are available in Table S1. CCLE and GDSC lung SCC cancer cell lines molecular data was obtained from https://depmap.org and https://www.cancerrxgene.org/gdsc1000/GDSC1000_WebResources/Home.html, respectively. Specific cell line identifiers and sources are detailed in the Supporting Information. GDSC, CTRPv2 and PRISM studies drug sensitivity data were available within the *PharmacoGx* R package. Lung SCC CPTAC‐3 study gene expression and copy number alterations data were downloaded from the Supporting Information.^5^
