# Supplementary material for: Transcriptional profiling of molecular pathways allows for the definition of robust lung squamous cell carcinoma molecular subtypes with specific vulnerabilities
Source: Clin Transl Med. 2023 Sep 21;13(9):e1413. doi: 10.1002/ctm2.1413 (PMC10514261; doi:10.1002/ctm2.1413)
Supplement: Supplementary file 1 — Supporting Information [file CTM2-13-e1413-s002.docx]

SUPPLEMENTARY FIGURES

**Figure S1.** Flow diagram of gene expression datasets with search and filtering criteria for this study.


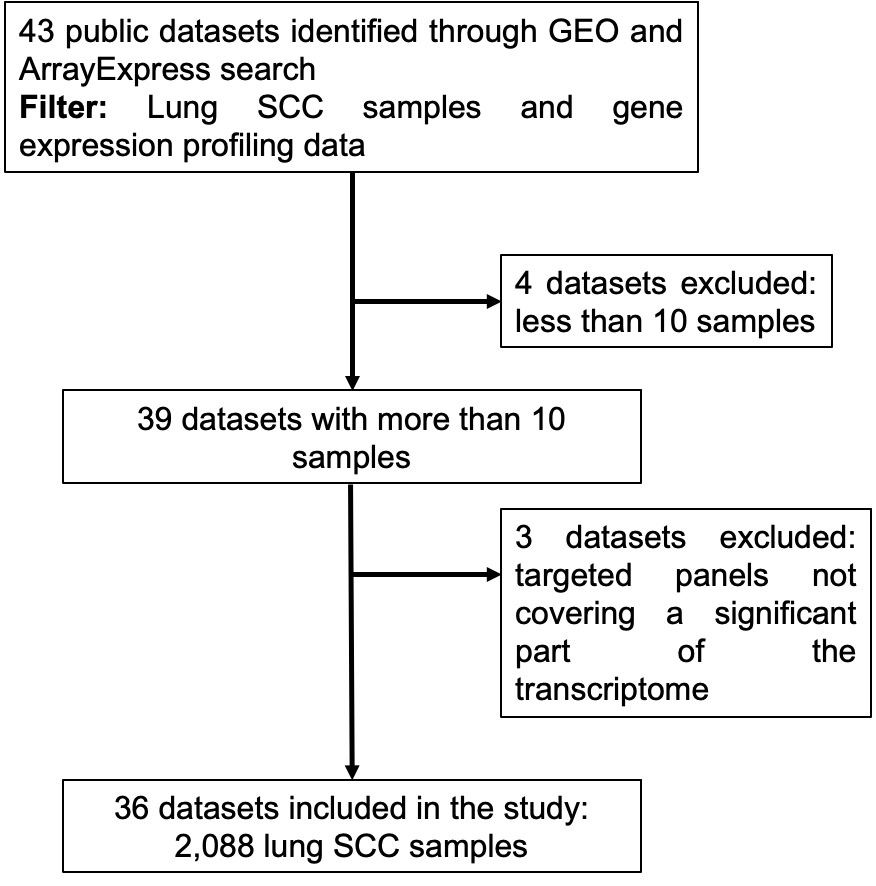


## Figure S2. Computational framework for definition of SCC consensus subtypes based on the evaluation of 50 molecular pathways at the transcriptional level.

### UMAP algorithm and walktrap graph-based clustering tool with Euclidean distance were used on each of the 500 GSVA scores matrices for SCC subpopulations identification. As a result, 500 potential classifications with different SCC subpopulations were obtained. From here, we conducted a series of steps subsequently enumerated: 1) mean GSVA scores for each evaluated pathway were calculated for the subpopulations found within each classification. We called this summary metrics *centroids*; 2) UMAP and walktrap method with Euclidean distance were applied to these centroids and consensus subtypes were identified; 3) Samples assigned to centroids belonging to different consensus subtypes were allocated to the subtype to which they had been assigned the majority of times across the different classifications. If a tie existed between two consensus subtypes, the sample was eliminated from subsequent analyses. In this case two samples were filtered out as a subtype could not be assigned. Thus, the final number of SCC samples with an assigned consensus subtype after this classification process is 2,086.


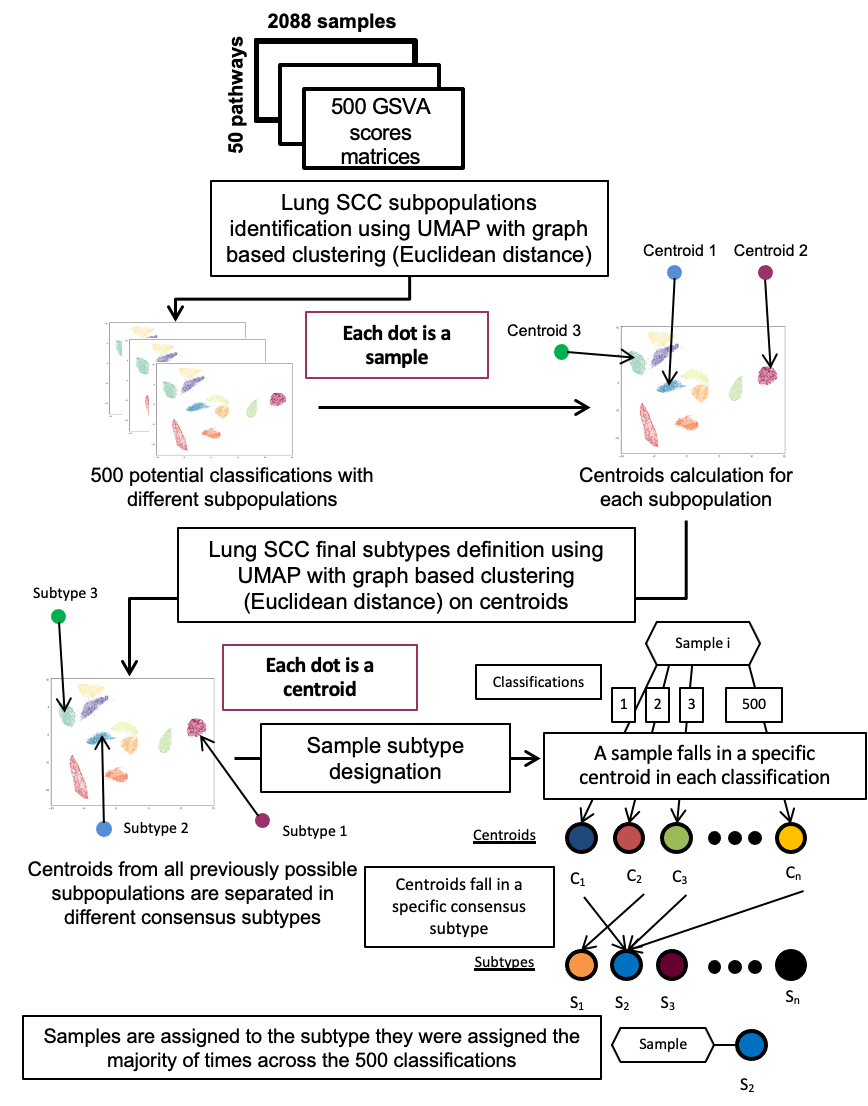


**Figure S3. Relative activity levels (GSVA scores) of the 50 studied landmark pathways across SCC subtypes.** U Mann Whitney test was used to perform pairwise comparisons for each molecular pathway. *P* values were corrected using Bonferroni multiple-testing correction method. (* p < 0.05 all pairwise comparisons, ** p < 0.01 all pairwise comparisons, *** p < 0.001 all pairwise comparisons, # p < 0.05 in three pairwise comparisons, ## p < 0.01 in three pairwise comparisons, ### p < 0.001 in three pairwise comparisons. Symbols on top or bottom of the boxplots represent upregulated or downregulated pathways in a specific subtype, respectively. Boxplots indicate median IQRs.


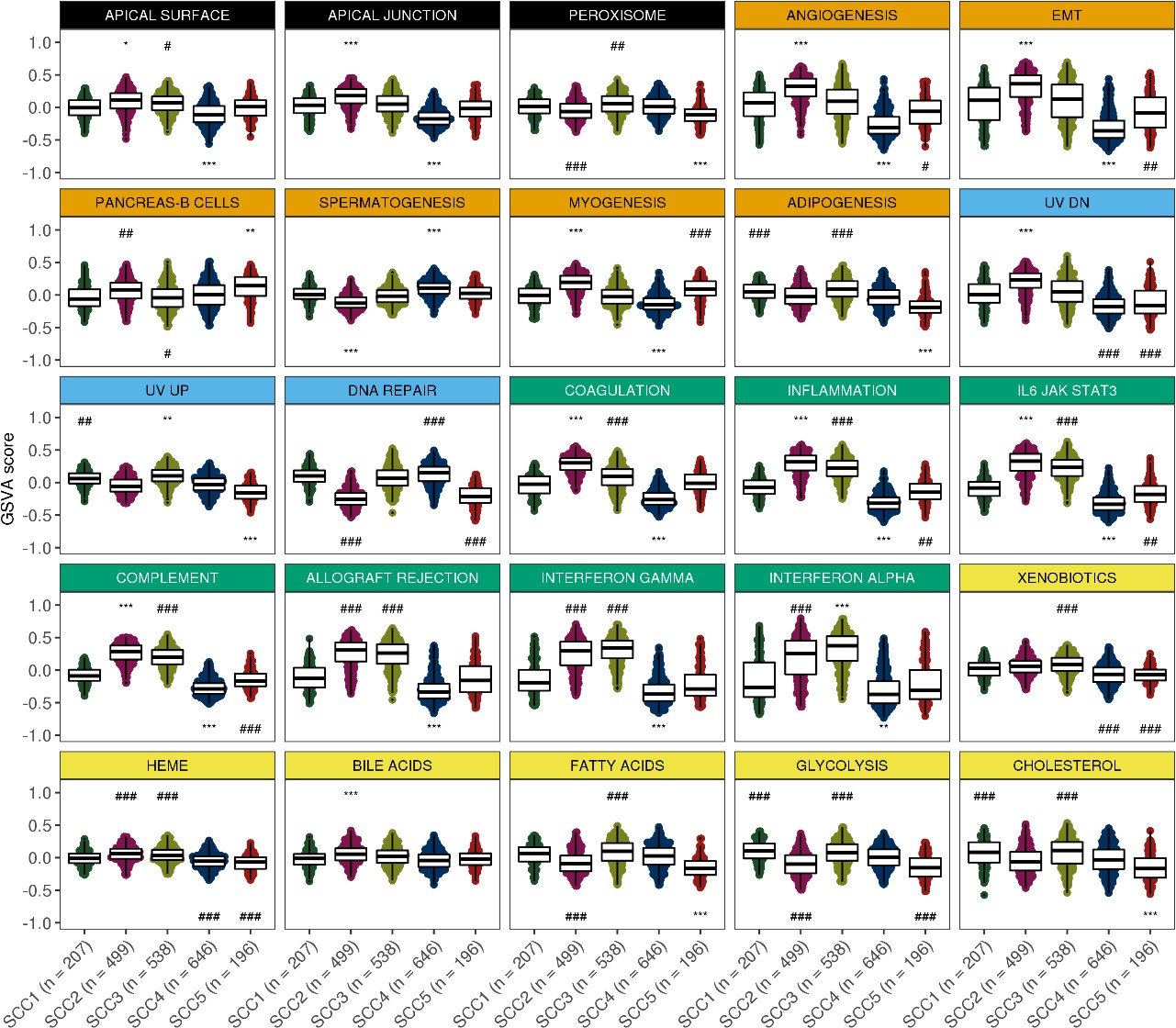


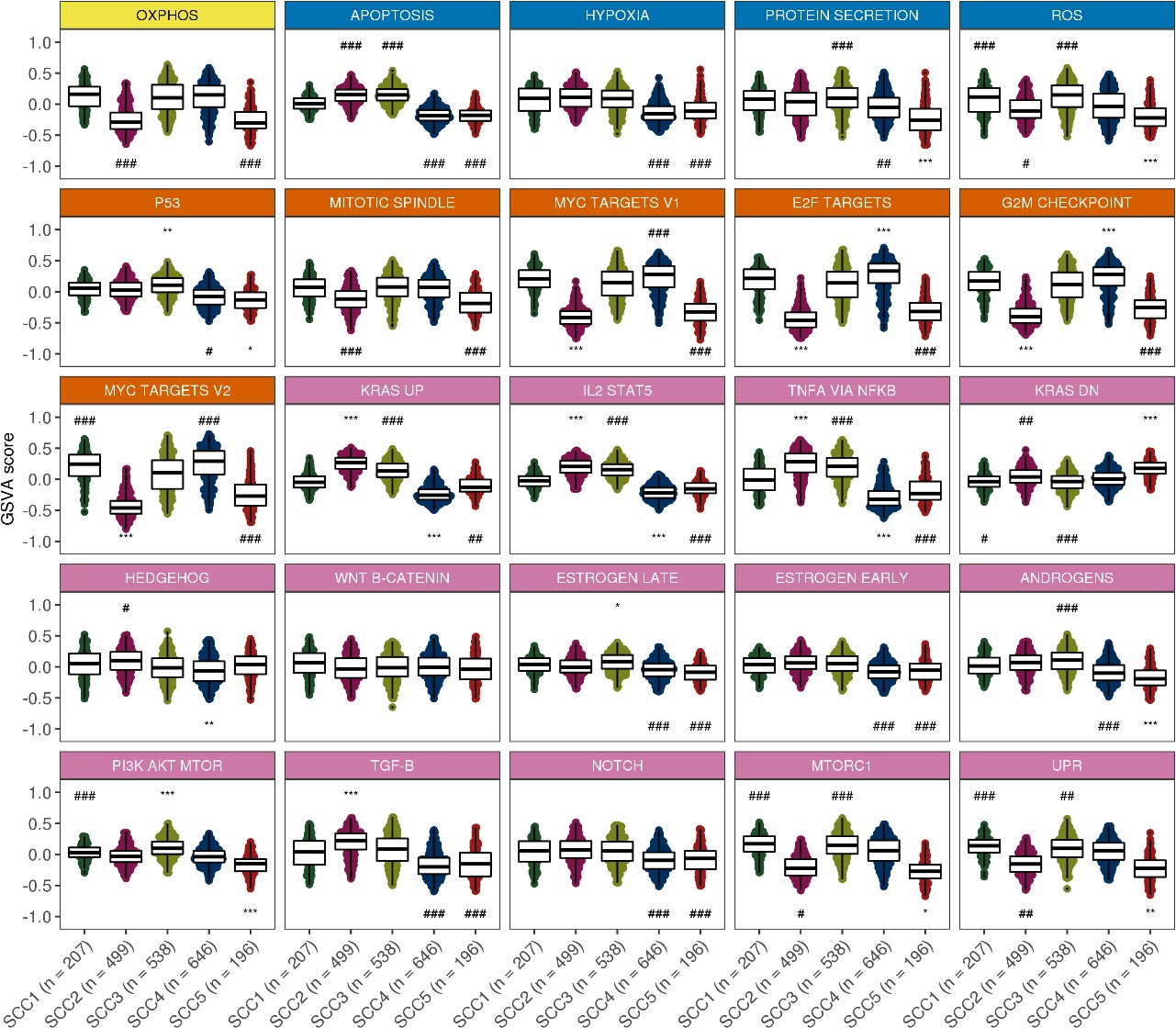


**Figure S4. Links between pathway profiling-based subtypes and Wilkerson et al.’s mRNA based subtypes. (A)** Wilkerson et al.’s SCC mRNA-based subtypes (i.e., secretory, basal, classical, and primitive) were assigned to each SCC sample using the nearest centroid predictor. SCC consensus pathway-based subtypes that best align with each Wilkerson et al. subtypes are displayed in each case, as well as the corresponding overlap value. **(B)** Overlap degree between the two classifications.

####
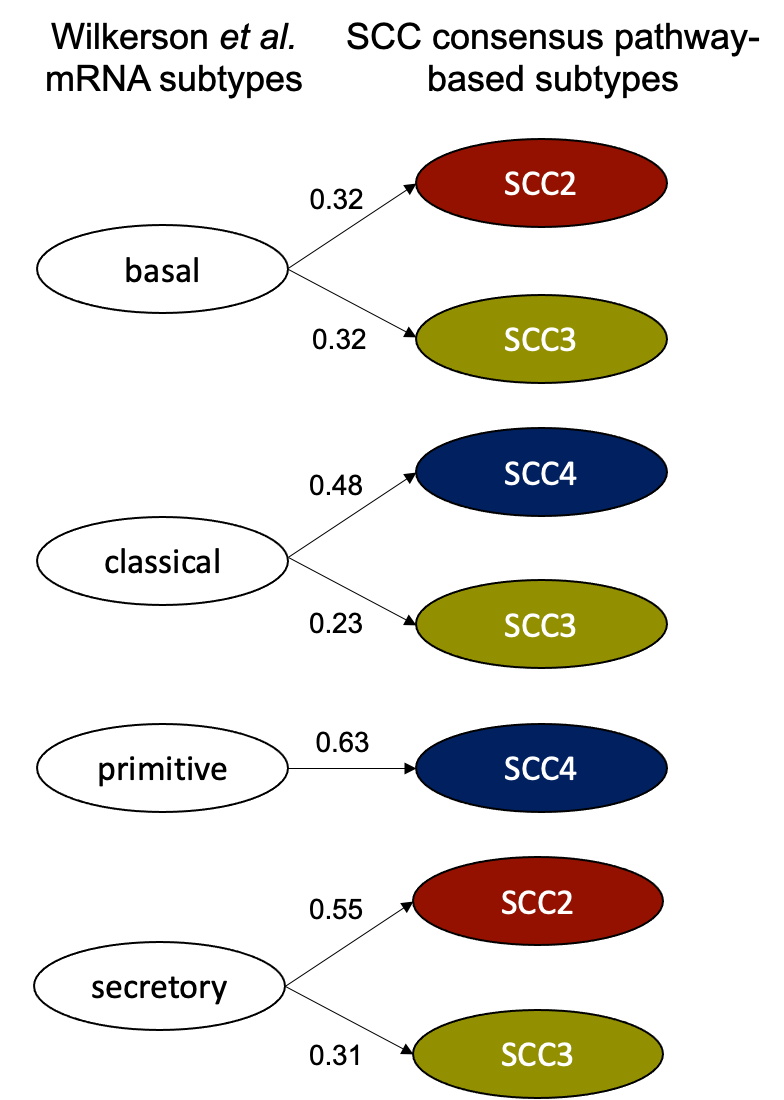
A

**B**


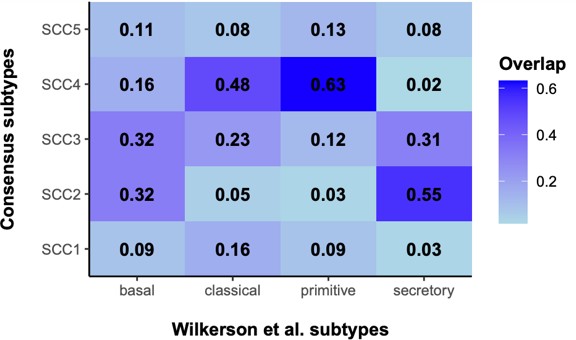


## Figure S5. Overall survival by lung SCC subtype. HR (95%-CI) come from a Cox proportional-hazards model adjusted for age, sex, stage, smoking history and study.

##
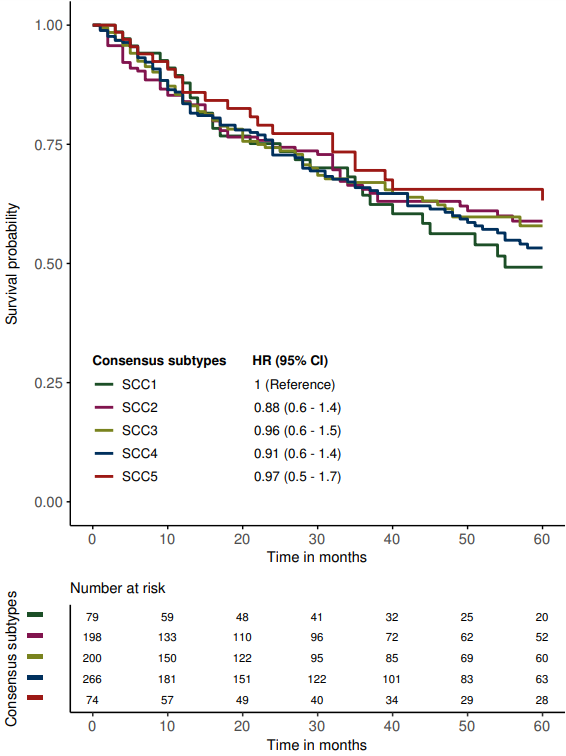


## Figure S6. Mean number of mutations associated with each identified COSMIC mutational signature across lung SCC subtypes.

##
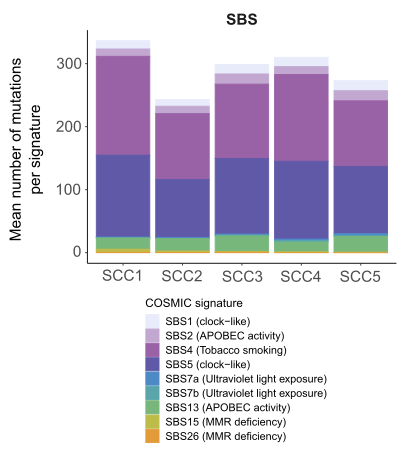


## Figure S7. Immune cell lines relative abundance across SCC subtypes.

U Mann Whitney test was used to perform pairwise comparisons for each immune population. *P* values were corrected using Bonferroni multiple-testing correction method. (* p < 0.05 all pairwise comparisons, ** p < 0.01 all pairwise comparisons, *** p < 0.001 all pairwise comparisons, # p < 0.05 in three pairwise comparisons, ## p < 0.01 in three pairwise comparisons, ### p < 0.001 in three pairwise comparisons. Symbols on top or bottom of the boxplots represent upregulated or downregulated pathways in a specific subtype, respectively. Boxplots indicate median and IQRs.


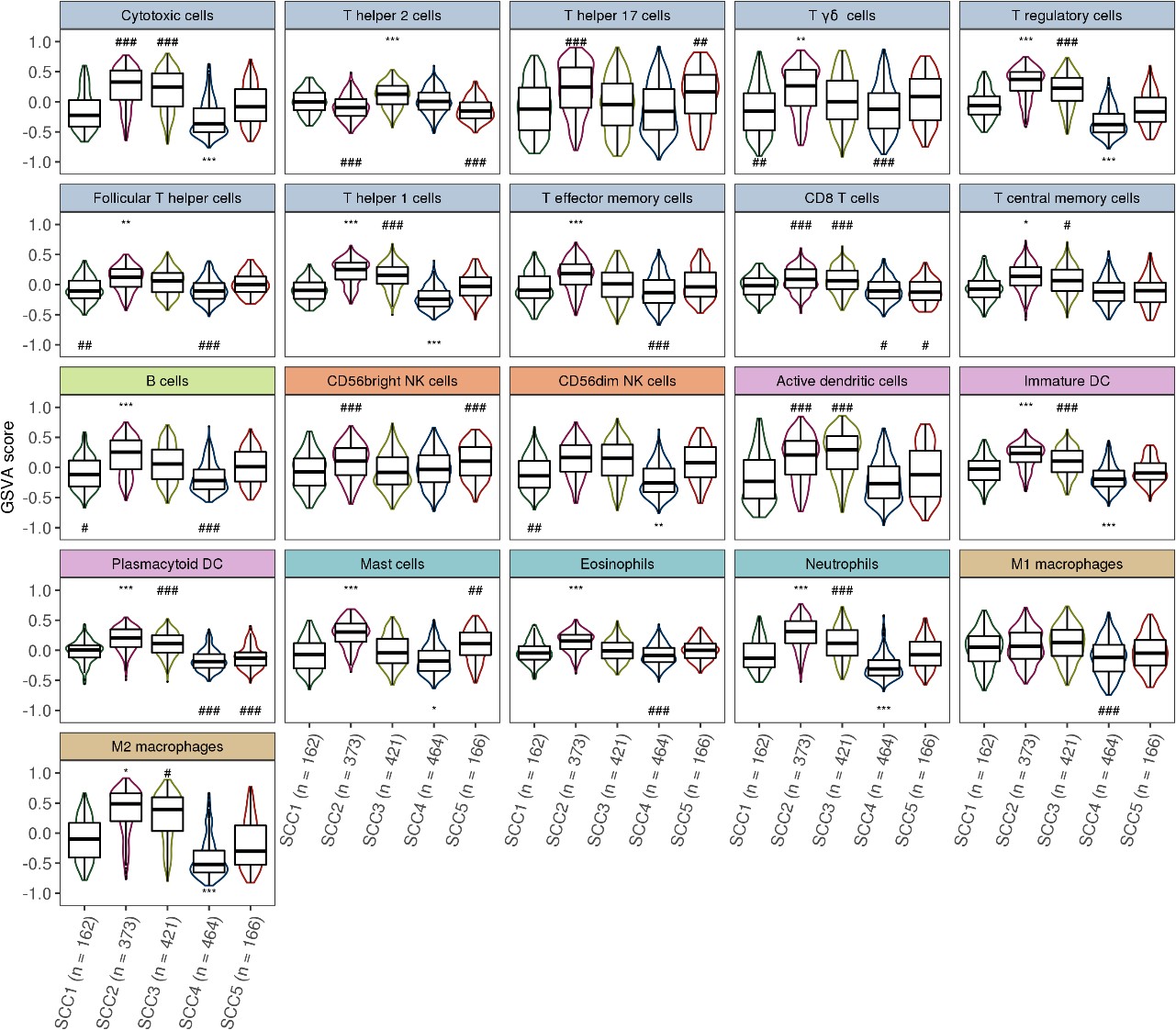


## Figure S8. Immune checkpoints expression. (Next page)

Expression of immune checkpoints across SCC subtypes per dataset. Genes are plotted whenever available in each sequencing platform. Datasets are ordered in decreasing order based on the sample size.

# CD274


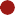

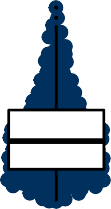

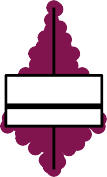

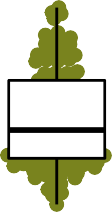

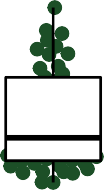

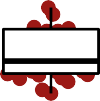


*p* = 3.5e−09

15

# PDCD1LG2

15


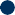

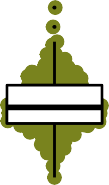

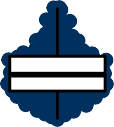

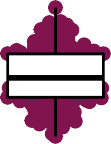

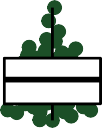

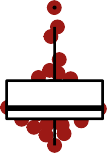


*p* = 6.5e−25

# PDCD1

15


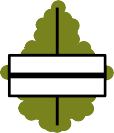

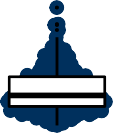

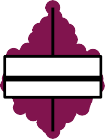

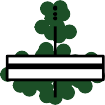

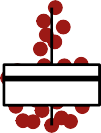

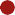


*p* = 1.4e−22

TCGA−LUSC(N = 500)

# CTLA4


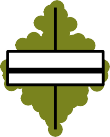

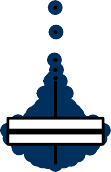

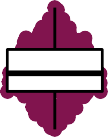

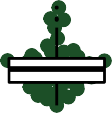

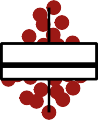

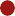


*p* = 6e−27

15

# HAVCR2

15

10 10 10 10 10

Expression

Expression

Expression

Expression

Expression

5 5 5 5 5

0 0 0 0 0

# LAG3


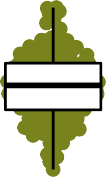

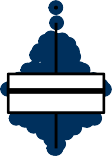

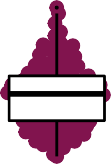

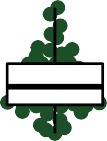

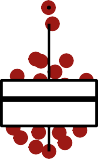


*p* = 4.5e−20

15

# TIGIT

15


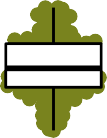

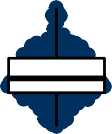

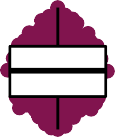

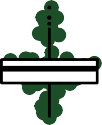

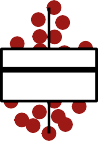

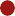


*p* = 2.2e−21

# BTLA

15


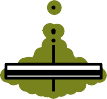

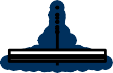

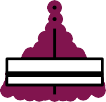

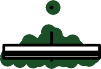

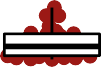

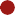


*p* = 2.7e−20

# IDO1

15

10 10 10 10

Expression

Expression

Expression

Expression

5 5 5 5

0 0 0 0

# CD276


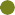

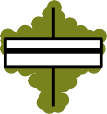

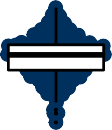

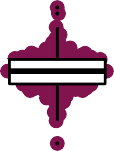

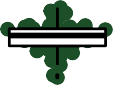

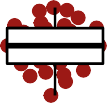

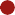


*p* = 0.07

15

# VTCN1

15


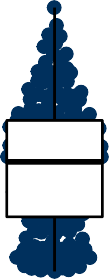

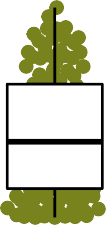

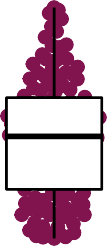

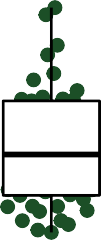

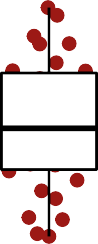


*p* = 0.013

*GEM*

15


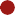

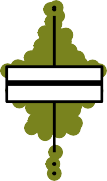

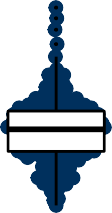

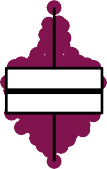

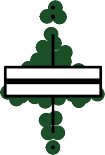

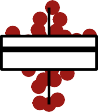


*p* = 1.1e−29


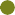

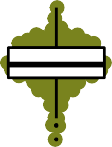

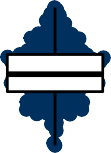

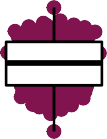

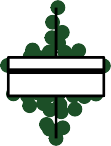

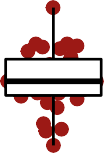


*p* = 5.4e−44


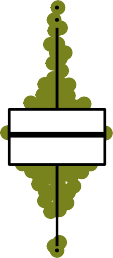

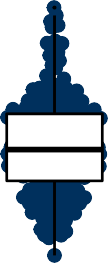

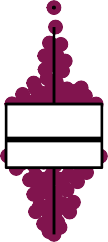

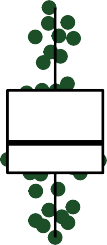

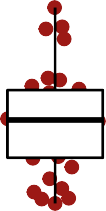


*p* = 4.4e−21

10 10 10

Expression

Expression

Expression

5 5 5

0 0 0

# PDCD1LG2


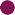

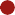

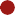

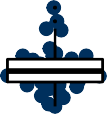

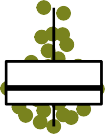

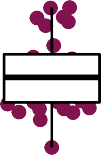


*p* = 0.0029

15

# PDCD1

15


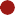

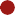

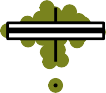

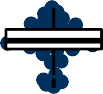

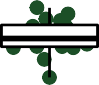

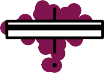


*p* = 0.0089

# CTLA4

15


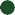

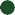

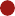

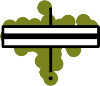

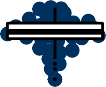

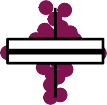

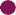


*p* = 0.0012

10 10 10

Expression

Expression

Expression

5 5 5

0 0 0

# LAG3


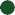

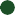


*p* = 0.0089

15

# IDO1

15

10 10

Expression

Expression

5 5

0 0

# VTCN1

*p* = 0.0082

15

*GEM*

15

*p* = 0.00011

*p* = 0.0049

10 10

Expression

Expression

5 5

0 0

# CD274

*p* = 0.0013

15

# PDCD1LG2

15

*p* = 2.3e−06

# PDCD1

15

*p* = 3.8e−06

# CTLA4

15

*p* = 1.9e−08

# HAVCR2

15

10 10 10 10 10

Expression

Expression

Expression

Expression

Expression

5 5 5 5 5

0 0 0 0 0

# LAG3

*p* = 0.02

15

# TIGIT

15

*p* = 6.6e−06

# VSIR

15

*p* = 7e−07

# BTLA

15

*p* = 4.7e−07

# IDO1

15

10 10 10 10 10

Expression

Expression

Expression

Expression

Expression

5 5 5 5 5

0 0 0 0 0

# CD276

*p* = 0.56

15

# VTCN1

15

*p* = 0.079

*GEM*

15

*p* = 1.4e−05

*p* = 8.2e−09

*p* = 0.00051

10 10 10

Expression

Expression

Expression

5 5 5

0 0 0

# CD274

*p* = 0.0036

15

# PDCD1LG2

15

*p* = 0.0032

# PDCD1

15

*p* = 0.42

# CTLA4

15

*p* = 0.0027

# HAVCR2

15

10 10 10 10 10

Expression

Expression

Expression

Expression

Expression

5 5 5 5 5

0 0 0 0 0

# LAG3

*p* = 8.3e−05

15

# TIGIT

15

*p* = 4e−05

# VSIR

15

*p* = 5.7e−07

# BTLA

15

*p* = 5.7e−05

# IDO1

15

10 10 10 10 10

Expression

Expression

Expression

Expression

Expression

5 5 5 5 5

0 0 0 0 0

# CD276

*p* = 0.46

15

# VTCN1

15

*p* = 0.86

*GEM*

15

*p* = 3.9e−06

*p* = 2.2e−07

*p* = 3.2e−06

10 10 10

Expression

Expression

Expression

5 5 5

0 0 0

# CD274

*p* = 1.3e−07

15

# PDCD1LG2

15

*p* = 0.12

# PDCD1

15

*p* = 5.1e−08

# CTLA4

15

*p* = 2.8e−05

# HAVCR2

15

10 10 10 10 10

Expression

Expression

Expression

Expression

Expression

5 5 5 5 5

0 0 0 0 0

# LAG3

*p* = 5.3e−07

15

# TIGIT

15

*p* = 5.1e−08

# BTLA

15

*p* = 1.5e−05

# IDO1

15

10 10 10 10

Expression

Expression

Expression

Expression

5 5 5 5

0 0 0 0

# CD276

*p* = 0.0027

15

# VTCN1

15

*p* = 0.66

*GEM*

15

*p* = 2.8e−07

*p* = 1.6e−08

*p* = 1.7e−07

10 10 10

Expression

Expression

Expression

5 5 5

0 0 0

# CD274

*p* = 0.00022

15

# PDCD1LG2

15

*p* = 0.00055

# PDCD1

15

*p* = 0.37

# CTLA4

15

*p* = 0.00053

# HAVCR2

15

10 10 10 10 10

Expression

Expression

Expression

Expression

Expression

5 5 5 5 5

0 0 0 0 0

# LAG3

*p* = 1e−05

15

# TIGIT

15

*p* = 3.1e−05

# VSIR

15

*p* = 5.9e−07

# BTLA

15

*p* = 5.6e−07

# IDO1

15

10 10 10 10 10

Expression

Expression

Expression

Expression

Expression

5 5 5 5 5

0 0 0 0 0

# CD276

*p* = 0.46

15

# VTCN1

15

*p* = 0.62

*GEM*

15

*p* = 1e−06

*p* = 1.7e−08

*p* = 1e−06

10 10 10

Expression

Expression

Expression

5 5 5

0 0 0

# CD274

*p* = 0.044

15

# PDCD1LG2

15

*p* = 0.073

# PDCD1

15

*p* = 0.00012

# CTLA4

15

*p* = 0.00033

# HAVCR2

15

10 10 10 10 10

Expression

Expression

Expression

Expression

Expression

5 5 5 5 5

0 0 0 0 0

# LAG3

*p* = 0.00043

15

# TIGIT

15

*p* = 0.0013

# VSIR

15

*p* = 5.6e−05

# BTLA

15

*p* = 8.9e−05

# IDO1

15

10 10 10 10 10

Expression

Expression

Expression

Expression

Expression

5 5 5 5 5

0 0 0 0 0

# CD276

*p* = 0.86

15

# VTCN1

15

*p* = 0.11

*GEM*

15

*p* = 4.1e−05

*p* = 3.2e−07

*p* = 0.00043

10 10 10

Expression

Expression

Expression

5 5 5

0 0 0

# CD274

*p* = 0.29

15

# PDCD1LG2

15

*p* = 0.13

# PDCD1

15

*p* = 0.014

# CTLA4

15

*p* = 0.2

# HAVCR2

15

10 10 10 10 10

Expression

Expression

Expression

Expression

Expression

5 5 5 5 5

0 0 0 0 0

# LAG3

*p* = 0.048

15

# TIGIT

15

*p* = 0.13

# VSIR

15

*p* = 2.9e−06

# BTLA

15

*p* = 0.0044

# IDO1

15

10 10 10 10 10

Expression

Expression

Expression

Expression

Expression

5 5 5 5 5

0 0 0 0 0

# CD276

*p* = 0.048

15

# VTCN1

15

*p* = 0.13

*GEM*

15

*p* = 0.00019

*p* = 2.1e−05

*p* = 0.0025

10 10 10

Expression

Expression

Expression

5 5 5

0 0 0

# CD274

*p* = 0.24

15

# PDCD1LG2

15

*p* = 0.41

# PDCD1

15

*p* = 0.36

# CTLA4

15

*p* = 1.8e−06

# HAVCR2

15

10 10 10 10 10

Expression

Expression

Expression

Expression

Expression

5 5 5 5 5

0 0 0 0 0

# LAG3

*p* = 3.1e−05

15

# TIGIT

15

*p* = 1.8e−06

# VSIR

15

*p* = 2.2e−08

# BTLA

15

*p* = 1.9e−06

# IDO1

15

10 10 10 10 10

Expression

Expression

Expression

Expression

Expression

5 5 5 5 5

0 0 0 0 0

# CD276

*p* = 0.025

15

# VTCN1

15

*p* = 0.42

*GEM*

15

*p* = 0.00038

*p* = 7.4e−08

*p* = 3.1e−05

10 10 10

Expression

Expression

Expression

5 5 5

0 0 0

# CD274

*p* = 0.35

15

# PDCD1LG2

15

*p* = 0.0015

# PDCD1

15

*p* = 7.7e−05

# CTLA4

15

*p* = 0.017

# HAVCR2

15

10 10 10 10 10

Expression

Expression

Expression

Expression

Expression

5 5 5 5 5

0 0 0 0 0

# LAG3

*p* = 0.00058

15

# TIGIT

15

*p* = 7.7e−05

# BTLA

15

*p* = 0.00076

# IDO1

15

10 10 10 10

Expression

Expression

Expression

Expression

5 5 5 5

0 0 0 0

# CD276

*p* = 0.3

15

# VTCN1

15

*p* = 0.28

*GEM*

15

*p* = 0.35

*p* = 2.5e−06

*p* = 0.00072

10 10 10

Expression

Expression

Expression

5 5 5

0 0 0

# CD274

*p* = 0.37

15

# PDCD1LG2

15

*p* = 0.32

# PDCD1

15

*p* = 0.77

# CTLA4

15

*p* = 0.051

# HAVCR2

15

10 10 10 10 10

Expression

Expression

Expression

Expression

Expression

5 5 5 5 5

0 0 0 0 0

# LAG3

*p* = 0.02

15

# TIGIT

15

*p* = 0.019

# VSIR

15

*p* = 0.00032

# BTLA

15

*p* = 0.32

# IDO1

15

10 10 10 10 10

Expression

Expression

Expression

Expression

Expression

5 5 5 5 5

0 0 0 0 0

# CD276

*p* = 0.14

15

# VTCN1

15

*p* = 0.77

*GEM*

15

*p* = 0.0033

*p* = 0.0013

*p* = 0.027

10 10 10

Expression

Expression

Expression

5 5 5

0 0 0

# CD274

*p* = 0.36

15

# PDCD1LG2

15

*p* = 0.81

# PDCD1

15

*p* = 0.0031

# CTLA4

15

*p* = 0.0031

# HAVCR2

15

10 10 10 10 10

Expression

Expression

Expression

Expression

Expression

5 5 5 5 5

0 0 0 0 0

# LAG3

*p* = 0.0071

15

# TIGIT

15

*p* = 0.07

# VSIR

15

*p* = 0.0031

# BTLA

15

*p* = 0.061

# IDO1

15

10 10 10 10 10

Expression

Expression

Expression

Expression

Expression

5 5 5 5 5

0 0 0 0 0

# CD276

*p* = 0.29

15

# VTCN1

15

*p* = 0.24

*GEM*

15

*p* = 0.02

*p* = 0.0031

*p* = 0.0031

10 10 10

Expression

Expression

Expression

5 5 5

0 0 0

# CD274

*p* = 0.039

15

# PDCD1LG2

15

*p* = 0.014

# PDCD1

15

*p* = 0.26

# CTLA4

15

*p* = 0.59

# HAVCR2

15

10 10 10 10 10

Expression

Expression

Expression

Expression

Expression

5 5 5 5 5

0 0 0 0 0

# LAG3

*p* = 0.022

15

# TIGIT

15

*p* = 0.014

# VSIR

15

*p* = 0.0011

# BTLA

15

*p* = 0.058

# IDO1

15

10 10 10 10 10

Expression

Expression

Expression

Expression

Expression

5 5 5 5 5

0 0 0 0 0

# CD276

*p* = 0.039

15

# VTCN1

15

*p* = 0.59

*GEM*

15

*p* = 0.0071

*p* = 0.01

*p* = 0.058

10 10 10

Expression

Expression

Expression

5 5 5

0 0 0

# PDCD1LG2

*p* = 0.079

15

# PDCD1

15

*p* = 0.0033

# CTLA4

15

*p* = 0.0033

10 10 10

Expression

Expression

Expression

5 5 5

0 0 0

# LAG3

*p* = 0.026

15

# IDO1

15

10 10

Expression

Expression

5 5

0 0

# VTCN1

*p* = 0.62

15

*GEM*

15

*p* = 0.012

*p* = 0.015

10 10

Expression

Expression

5 5

0 0

# CD274

*p* = 0.0083

15

# PDCD1LG2

15

*p* = 0.033

# PDCD1

15

*p* = 0.54

# CTLA4

15

*p* = 0.87

# HAVCR2

15

10 10 10 10 10

Expression

Expression

Expression

Expression

Expression

5 5 5 5 5

0 0 0 0 0

# LAG3

*p* = 0.015

15

# TIGIT

15

*p* = 0.55

# BTLA

15

*p* = 0.056

# IDO1

15

10 10 10 10

Expression

Expression

Expression

Expression

5 5 5 5

0 0 0 0

# CD276

*p* = 0.2

15

# VTCN1

15

*p* = 0.54

*GEM*

15

*p* = 0.011

*p* = 0.00049

*p* = 0.24

10 10 10

Expression

Expression

Expression

5 5 5

0 0 0

# CD274

*p* = 0.11

15

# PDCD1LG2

15

*p* = 0.17

# PDCD1

15

*p* = 0.0046

# CTLA4

15

*p* = 0.017

# HAVCR2

15

10 10 10 10 10

Expression

Expression

Expression

Expression

Expression

5 5 5 5 5

0 0 0 0 0

# LAG3

*p* = 0.0069

15

# TIGIT

15

*p* = 0.014

# VSIR

15

*p* = 0.0046

# BTLA

15

*p* = 0.0084

# IDO1

15

10 10 10 10 10

Expression

Expression

Expression

Expression

Expression

5 5 5 5 5

0 0 0 0 0

# CD276

*p* = 0.72

15

# VTCN1

15

*p* = 0.19

*GEM*

15

*p* = 0.014

*p* = 0.00038

*p* = 0.026

10 10 10

Expression

Expression

Expression

5 5 5

0 0 0

# CD274

*p* = 0.19

15

# PDCD1LG2

15

*p* = 0.95

# PDCD1

15

*p* = 0.018

# CTLA4

15

*p* = 0.19

# HAVCR2

15

10 10 10 10 10

Expression

Expression

Expression

Expression

Expression

5 5 5 5 5

0 0 0 0 0

# LAG3

*p* = 0.31

15

# TIGIT

15

*p* = 0.017

# VSIR

15

*p* = 0.0032

# BTLA

15

*p* = 0.19

# IDO1

15

10 10 10 10 10

Expression

Expression

Expression

Expression

Expression

5 5 5 5 5

0 0 0 0 0

# CD276

*p* = 0.19

15

# VTCN1

15

*p* = 0.81

*GEM*

15

*p* = 0.0067

*p* = 0.14

*p* = 0.29

10 10 10

Expression

Expression

Expression

5 5 5

0 0 0

# CD274

*p* = 0.46

15

# PDCD1LG2

15

*p* = 0.33

# PDCD1

15

*p* = 0.026

# CTLA4

15

*p* = 0.084

# HAVCR2

15

10 10 10 10 10

Expression

Expression

Expression

Expression

Expression

5 5 5 5 5

0 0 0 0 0

# LAG3

*p* = 0.038

15

# TIGIT

15

*p* = 0.017

# BTLA

15

*p* = 0.021

# IDO1

15

10 10 10 10

Expression

Expression

Expression

Expression

5 5 5 5

0 0 0 0

# CD276

*p* = 0.096

15

# VTCN1

15

*p* = 0.94

*GEM*

15

*p* = 0.0071

*p* = 0.0071

*p* = 0.21

10 10 10

Expression

Expression

Expression

5 5 5

0 0 0

# CD274

*p* = 0.096

15

# PDCD1LG2

15

*p* = 0.22

# PDCD1

15

*p* = 0.11

# CTLA4

15

*p* = 0.064

# HAVCR2

15

10 10 10 10 10

Expression

Expression

Expression

Expression

Expression

5 5 5 5 5

0 0 0 0 0

# LAG3

*p* = 0.21

15

# TIGIT

15

*p* = 0.016

# VSIR

15

*p* = 6e−04

# BTLA

15

*p* = 0.1

# IDO1

15

10 10 10 10 10

Expression

Expression

Expression

Expression

Expression

5 5 5 5 5

0 0 0 0 0

# CD276

*p* = 0.56

15

# VTCN1

15

*p* = 0.64

*GEM*

15

*p* = 0.052

*p* = 0.0027

*p* = 0.0098

10 10 10

Expression

Expression

Expression

5 5 5

0 0 0

# CD274

*p* = 0.58

15

# PDCD1LG2

15

*p* = 0.22

# PDCD1

15

*p* = 0.15

# CTLA4

15

*p* = 0.02

# HAVCR2

15

10 10 10 10 10

Expression

Expression

Expression

Expression

Expression

5 5 5 5 5

0 0 0 0 0

# LAG3

*p* = 0.22

15

# BTLA

15

*p* = 0.02

10 10

Expression

Expression

5 5

0 0

# CD276

*p* = 0.24

15

# VTCN1

15

*p* = 0.69

*GEM*

15

*p* = 0.15

*p* = 0.0027

10 10 10

Expression

Expression

Expression

5 5 5

0 0 0

# CD274

*p* = 0.48

15

# PDCD1LG2

15

*p* = 0.11

# PDCD1

15

*p* = 0.67

# CTLA4

15

*p* = 0.43

# HAVCR2

15

10 10 10 10 10

Expression

Expression

Expression

Expression

Expression

5 5 5 5 5

0 0 0 0 0

# LAG3

*p* = 0.38

15

# TIGIT

15

*p* = 0.024

# VSIR

15

*p* = 0.015

# BTLA

15

*p* = 0.35

# IDO1

15

10 10 10 10 10

Expression

Expression

Expression

Expression

Expression

5 5 5 5 5

0 0 0 0 0

# CD276

*p* = 0.48

15

# VTCN1

15

*p* = 0.48

*GEM*

15

*p* = 0.43

*p* = 0.023

*p* = 0.17

10 10 10

Expression

Expression

Expression

5 5 5

0 0 0

# CD274

*p* = 0.93

15

# PDCD1LG2

15

*p* = 0.84

# PDCD1

15

*p* = 0.84

# CTLA4

15

*p* = 0.44

# HAVCR2

15

10 10 10 10 10

Expression

Expression

Expression

Expression

Expression

5 5 5 5 5

0 0 0 0 0

# LAG3

*p* = 0.93

15

# TIGIT

15

*p* = 0.38

# VSIR

15

*p* = 0.31

# BTLA

15

*p* = 0.44

# IDO1

15

10 10 10 10 10

Expression

Expression

Expression

Expression

Expression

5 5 5 5 5

0 0 0 0 0

# CD276

*p* = 0.35

15

# VTCN1

15

*p* = 0.93

*GEM*

15

*p* = 0.38

*p* = 0.35

*p* = 0.69

10 10 10

Expression

Expression

Expression

5 5 5

0 0 0

# CD274

*p* = 0.52

15

# PDCD1LG2

15

*p* = 0.51

# PDCD1

15

*p* = 0.52

# CTLA4

15

*p* = 0.52

# HAVCR2

15

10 10 10 10 10

Expression

Expression

Expression

Expression

Expression

5 5 5 5 5

0 0 0 0 0

# LAG3

*p* = 0.52

15

# TIGIT

15

*p* = 0.52

# VSIR

15

*p* = 0.15

# BTLA

15

*p* = 0.51

# IDO1

15

10 10 10 10 10

Expression

Expression

Expression

Expression

Expression

5 5 5 5 5

0 0 0 0 0

# CD276

*p* = 0.52

15

# VTCN1

15

*p* = 0.79

*GEM*

15

*p* = 0.15

*p* = 0.38

*p* = 0.52

10 10 10

Expression

Expression

Expression

5 5 5

0 0 0

# CD274

*p* = 0.23

15

# PDCD1LG2

15

*p* = 0.19

# CTLA4

15

*p* = 0.19

# HAVCR2

15

10 10 10 10

Expression

Expression

Expression

Expression

5 5 5 5

0 0 0 0

# LAG3

*p* = 0.23

15

# TIGIT

15

*p* = 0.21

# VSIR

15

*p* = 0.19

# BTLA

15

*p* = 0.64

# IDO1

15

10 10 10 10 10

Expression

Expression

Expression

Expression

Expression

5 5 5 5 5

0 0 0 0 0

# CD276

*p* = 0.37

15

# VTCN1

15

*p* = 0.4

*GEM*

15

*p* = 0.19

*p* = 0.19

*p* = 0.4

10 10 10

Expression

Expression

Expression

5 5 5

0 0 0

# CD274

*p* = 0.61

15

# PDCD1LG2

15

*p* = 0.76

# PDCD1

15

*p* = 0.057

# CTLA4

15

*p* = 0.027

# HAVCR2

15

10 10 10 10 10

Expression

Expression

Expression

Expression

Expression

5 5 5 5 5

0 0 0 0 0

# LAG3

*p* = 0.057

15

# TIGIT

15

*p* = 0.067

# VSIR

15

*p* = 0.027

# BTLA

15

*p* = 0.057

# IDO1

15

10 10 10 10 10

Expression

Expression

Expression

Expression

Expression

5 5 5 5 5

0 0 0 0 0

# CD276

*p* = 0.61

15

# VTCN1

15

*p* = 0.76

*GEM*

15

*p* = 0.027

*p* = 0.027

*p* = 0.12

10 10 10

Expression

Expression

Expression

5 5 5

0 0 0

# CD274

*p* = 0.34

15

# PDCD1LG2

15

*p* = 0.16

# CTLA4

15

*p* = 0.059

10 10 10

Expression

Expression

Expression

5 5 5

0 0 0

# CD276

*p* = 0.98

15

# VTCN1

15

*p* = 0.39

# GEM

15

*p* = 0.39

10 10 10

Expression

Expression

Expression

5 5 5

0 0 0

# CD274

*p* = 0.59

15

# PDCD1LG2

15

*p* = 0.27

# PDCD1

15

*p* = 0.28

# CTLA4

15

*p* = 0.16

# HAVCR2

15

10 10 10 10 10

Expression

Expression

Expression

Expression

Expression

5 5 5 5 5

0 0 0 0 0

# LAG3

*p* = 0.56

15

# TIGIT

15

*p* = 0.31

# VSIR

15

*p* = 0.16

# BTLA

15

*p* = 0.27

# IDO1

15

10 10 10 10 10

Expression

Expression

Expression

Expression

Expression

5 5 5 5 5

0 0 0 0 0

# CD276

*p* = 0.16

15

# VTCN1

15

*p* = 0.28

*GEM*

15

*p* = 0.18

*p* = 0.16

*p* = 0.27

10 10 10

Expression

Expression

Expression

5 5 5

0 0 0

# PDCD1

*p* = 0.15

15

10

Expression

5

0

# LAG3

*p* = 0.15

15

# IDO1

15

*p* = 0.37

10 10

Expression

Expression

5 5

0 0

# GEM

*p* = 0.89

15

10

Expression

5

0

# PDCD1

*p* = 0.43

15

10

Expression

5

0

# LAG3

*p* = 0.43

15

# IDO1

15

*p* = 0.43

10 10

Expression

Expression

5 5

0 0

# GEM

*p* = 0.43

15

10

Expression

5

0

# CD274

*p* = 0.32

15

# PDCD1LG2

15

*p* = 0.24

# PDCD1

15

*p* = 0.32

# CTLA4

15

*p* = 0.32

# HAVCR2

15

10 10 10 10 10

Expression

Expression

Expression

Expression

Expression

5 5 5 5 5

0 0 0 0 0

# LAG3

*p* = 0.32

15

# TIGIT

15

*p* = 0.32

# VSIR

15

*p* = 0.16

# BTLA

15

*p* = 0.45

# IDO1

15

10 10 10 10 10

Expression

Expression

Expression

Expression

Expression

5 5 5 5 5

0 0 0 0 0

# CD276

*p* = 0.38

15

# VTCN1

15

*p* = 0.24

*GEM*

15

*p* = 0.16

*p* = 0.24

*p* = 0.24

10 10 10

Expression

Expression

Expression

5 5 5

0 0 0

# CD274

*p* = 0.3

15

# PDCD1LG2

15

*p* = 0.54

# PDCD1

15

*p* = 0.4

# CTLA4

15

*p* = 0.3

# HAVCR2

15

10 10 10 10 10

Expression

Expression

Expression

Expression

Expression

5 5 5 5 5

0 0 0 0 0

# LAG3

*p* = 0.4

15

# TIGIT

15

*p* = 0.4

# VSIR

15

*p* = 0.3

# BTLA

15

*p* = 0.4

# IDO1

15

10 10 10 10 10

Expression

Expression

Expression

Expression

Expression

5 5 5 5 5

0 0 0 0 0

# CD276

*p* = 0.54

15

# VTCN1

15

*p* = 0.54

*GEM*

15

*p* = 0.4

*p* = 0.4

*p* = 0.45

10 10 10

Expression

Expression

Expression

5 5 5

0 0 0

# PDCD1LG2

*p* = 0.46

15

# PDCD1

15

*p* = 0.46

# CTLA4

15

*p* = 0.69

10 10 10

Expression

Expression

Expression

5 5 5

0 0 0

# LAG3

*p* = 0.15

15

# IDO1

15

*p* = 0.15

10 10

Expression

Expression

5 5

0 0

# GEM

*p* = 0.46

15

10

Expression

5

0

# CD274

*p* = 0.3

15

# PDCD1LG2

15

*p* = 0.4

# PDCD1

15

*p* = 0.31

# CTLA4

15

*p* = 0.3

# HAVCR2

15

10 10 10 10 10

Expression

Expression

Expression

Expression

Expression

5 5 5 5 5

0 0 0 0 0

# LAG3

*p* = 0.3

15

# TIGIT

15

*p* = 0.3

# VSIR

15

*p* = 0.3

# BTLA

15

*p* = 0.36

# IDO1

15

10 10 10 10 10

Expression

Expression

Expression

Expression

Expression

5 5 5 5 5

0 0 0 0 0

# CD276

*p* = 0.46

15

# VTCN1

15

*p* = 0.7

*GEM*

15

*p* = 0.34

*p* = 0.3

*p* = 0.3

10 10 10

Expression

Expression

Expression

5 5 5

0 0 0

# CD274

*p* = 0.6

15

# PDCD1LG2

15

*p* = 0.37

# PDCD1

15

*p* = 0.6

# CTLA4

15

*p* = 0.6

# HAVCR2

15

10 10 10 10 10

Expression

Expression

Expression

Expression

Expression

5 5 5 5 5

0 0 0 0 0

# LAG3

*p* = 0.74

15

# TIGIT

15

*p* = 0.6

# VSIR

15

*p* = 0.37

# BTLA

15

*p* = 0.49

# IDO1

15

10 10 10 10 10

Expression

Expression

Expression

Expression

Expression

5 5 5 5 5

0 0 0 0 0

# CD276

*p* = 0.37

15

# VTCN1

15

*p* = 0.6

*GEM*

15

*p* = 0.37

*p* = 0.39

*p* = 0.37

10 10 10

Expression

Expression

Expression

5 5 5

0 0 0

# CD274

*p* = 0.31

15

# PDCD1LG2

15

*p* = 0.31

# PDCD1

15

*p* = 0.7

# CTLA4

15

*p* = 0.39

# HAVCR2

15

10 10 10 10 10

Expression

Expression

Expression

Expression

Expression

5 5 5 5 5

0 0 0 0 0

# LAG3

*p* = 0.39

15

# TIGIT

15

*p* = 0.39

# BTLA

15

*p* = 0.39

# IDO1

15

10 10 10 10

Expression

Expression

Expression

Expression

5 5 5 5

0 0 0 0

# CD276

*p* = 0.31

15

# VTCN1

15

*p* = 0.73

*GEM*

15

*p* = 0.39

*p* = 0.31

*p* = 0.31

10 10 10

Expression

Expression

Expression

5 5 5

0 0 0

# CD274

*p* = 0.47

15

# CTLA4

15

*p* = 0.47

10 10

Expression

Expression

5 5

0 0

# VSIR

*p* = 0.95

15

# BTLA

15

*p* = 0.69

# IDO1

15

10 10 10

Expression

Expression

Expression

5 5 5

0 0 0

# CD276

*p* = 0.69

15

# VTCN1

15

*p* = 0.69

*GEM*

15

*p* = 0.69

*p* = 0.69

10 10 10

Expression

Expression

Expression

5 5 5

0 0 0

## Figure S9. Assessment of gene expression signatures predictive of carboplatin and cisplatin resistance in the discovery and validation cohorts. Enrichment assessment for signatures of resistance to currently established first-line chemotherapy in patients with lung SCC (carboplatin—left panel, cisplatin—right panel) in the discovery (upper panels) and validation sets (bottom panels). Each lung SCC tumor is represented with a dot, and GSVA scores (y-axis) indicate the upregulation of the resistance signature in each patient (as indicated in each plot title).

##

## Figure S10. Assessment of discovery set derived subtype-specific gene expression signatures in the CPTAC-3 validation cohort. Each lung SCC tumor is represented with a dot, and GSVA scores (y-axis) indicate the presence of each signature in each patient.

##

## Figure S11. Lung SCC cancer cell lines (SCC-CCL) used for the potential treatment strategies discovery analysis.

### Subtype assignation along with genomic information available from CCLE project cell lines (CTRPv2 and PRISM drug sensitivity datasets).

1. Subtype assignation along with genomic information available from GDSC project cell lines (GDSC drug sensitivity datasets).

### Cell lines in each dataset were classified into the different SCC subtypes using the *predict* function of the UMAP R package based on the global behavior of the same 50 pathways used for the primary tumors’ classification. Genomic characteristics regarding important driver genes for NSCLC clinical management are displayed, when available. Green color represents the presence of a mutation in a specific gene. Cell line names in each subtype are named after their Cellosaurus ID.

#### B
