# Supplementary material for: Transcriptional profiling of molecular pathways allows for the definition of robust lung squamous cell carcinoma molecular subtypes with specific vulnerabilities
Source: Clin Transl Med. 2023 Sep 21;13(9):e1413. doi: 10.1002/ctm2.1413 (PMC10514261; doi:10.1002/ctm2.1413)
Supplement: Supplementary file 2 — Supporting Information [file CTM2-13-e1413-s001.docx]

**SUPPLEMENTARY TABLES**

**Table S1.** List of gene expression datasets included in this study.

| **Dataset ID** | **Paper ID** | **Resource** | **Platform** | **Number SCC samples** |
| --- | --- | --- | --- | --- |
| [GSE3141](https://www.ncbi.nlm.nih.gov/geo/query/acc.cgi?acc=GSE3141) | [16273092](https://www.ncbi.nlm.nih.gov/pubmed/16273092) | GEO (Bild) | Affymetrix HG-U133_Plus_2 | 53 |
| GSE12472 | [20832896](https://www.ncbi.nlm.nih.gov/pubmed/20832896) | GEO (Boelens) | Agilent-012391 Whole Human Genome Oligo Microarray G4112A | 34 |
| [GSE37745](https://www.ncbi.nlm.nih.gov/geo/query/acc.cgi?acc=GSE37745) | [23032747](https://www.ncbi.nlm.nih.gov/pubmed/23032747) | GEO (Botling) | Affymetrix HG-U133_Plus_2 | 66 |
| [GSE1987](https://www.ncbi.nlm.nih.gov/geo/query/acc.cgi?acc=GSE1987) | [17258348](https://www.ncbi.nlm.nih.gov/pubmed/17258348) | GEO (Dehan) | Affymetrix HG U95A Array | 17 |
| [GSE50081](https://www.ncbi.nlm.nih.gov/geo/query/acc.cgi?acc=GSE50081) | [24305008](https://www.ncbi.nlm.nih.gov/pubmed/24305008) | GEO (Der) | Affymetrix HG-U133_Plus_2 | 43 |
| [E-MTAB-1132](https://www.ebi.ac.uk/arrayexpress/experiments/E-MTAB-1132) | [25944621](https://www.ncbi.nlm.nih.gov/pubmed/25944621) | ArrayExpress (Lazar) | Agilent_Human_244K_Exonv3 | 50 |
| [E-MTAB-1727](https://www.ebi.ac.uk/arrayexpress/experiments/E-MTAB-1727) | [27197161](https://www.ncbi.nlm.nih.gov/pubmed/27197161) | ArrayExpress (Brambilla) | Affymetrix HG-U133_Plus_2 | 93 |
| [E-MTAB-1790](https://www.ebi.ac.uk/arrayexpress/experiments/E-MTAB-1790) | [25301404](https://www.ncbi.nlm.nih.gov/pubmed/25301404) | ArrayExpress (Sanz) | Agilent HG array 44k_014850 | 41 |
| [E-MTAB-5231](https://www.ebi.ac.uk/gxa/experiments/E-MTAB-5231?ref=aebrowse) | [28292941](https://www.ncbi.nlm.nih.gov/pubmed/28292941) | ArrayExpress (Joerg Mueller) | Affymetrix HG-U133_Plus_2 | 11 |
| [GSE2109](https://www.ncbi.nlm.nih.gov/geo/query/acc.cgi?acc=GSE2109) | - | GEO (expO) | Affymetrix HG-U133_Plus_2 | 40 |
| [GSE20853](https://www.ncbi.nlm.nih.gov/geo/query/acc.cgi?acc=GSE20853) | [21737174](https://www.ncbi.nlm.nih.gov/pubmed/21737174) | GEO (Fujiwara) | CHUGAI 41K (spotted DNA/cDNA) | 10 |
| [GSE31552](https://www.ncbi.nlm.nih.gov/geo/query/acc.cgi?acc=GSE31552) | [25128906](https://www.ncbi.nlm.nih.gov/pubmed/25128906) | GEO (Spivack) | Affymetrix HG 1.0 ST Gene Array | 11 |
| [GSE31799](https://www.ncbi.nlm.nih.gov/geo/query/acc.cgi?acc=GSE31799) | [21911935](https://www.ncbi.nlm.nih.gov/pubmed/21911935) | GEO (Starczynowski) | Affymetrix Custom Rosetta Human platform | 20 |
| [GSE33479](https://www.ncbi.nlm.nih.gov/geo/query/acc.cgi?acc=GSE33479) | - | GEO (Mascaux) | Agilent_44k_014850 | 95 |
| [GSE33532](https://www.ncbi.nlm.nih.gov/geo/query/acc.cgi?acc=GSE33532) | - | GEO (Meister) | Affymetrix HG-U133_Plus_2 | 16 |
| [GSE43580](https://www.ncbi.nlm.nih.gov/geo/query/acc.cgi?acc=GSE43580) | [23966112](https://www.ncbi.nlm.nih.gov/pubmed/23966112) | GEO (Tarca) | Affymetrix HG-U133_Plus_2 | 72 |
| [GSE6044](https://www.ncbi.nlm.nih.gov/geo/query/acc.cgi?acc=GSE6044) | [18992152](https://www.ncbi.nlm.nih.gov/pubmed/18992152) | GEO (Rohr) | Affymetrix Human HG-Focus Target Array | 14 |
| GSE60644 | [25278450](https://www.ncbi.nlm.nih.gov/pubmed/25278450) | GEO (Karlsson A) | Illumina HumanHT-12 V4.0 expression beadchip | 22 |
| [GSE6253](https://www.ncbi.nlm.nih.gov/geo/query/acc.cgi?acc=GSE6253) | [17194181](https://www.ncbi.nlm.nih.gov/pubmed/17194181) | GEO (Lu) | Affymetrix HG U95A Array | 18 |
| [GSE6253](https://www.ncbi.nlm.nih.gov/geo/query/acc.cgi?acc=GSE6253) | [17194181](https://www.ncbi.nlm.nih.gov/pubmed/17194181) | GEO (Lu) | Affymetrix HG-U133A | 18 |
| [GSE67061](https://www.ncbi.nlm.nih.gov/geo/query/acc.cgi?acc=GSE67061) | - | GEO (Tong) | Agilent_44k_014850 | 69 |
| [GSE19188](https://www.ncbi.nlm.nih.gov/geo/query/acc.cgi?acc=GSE19188) | [20421987](https://www.ncbi.nlm.nih.gov/pubmed/20421987) | GEO (Hou) | Affymetrix HG-U133_Plus_2 | 27 |
| [GSE10245](https://www.ncbi.nlm.nih.gov/geo/query/acc.cgi?acc=GSE10245) | [18486272](https://www.ncbi.nlm.nih.gov/pubmed/18486272) | GEO (Kuner) | Affymetrix HG-U133_Plus_2 | 14 |
| [GSE8894](https://www.ncbi.nlm.nih.gov/geo/query/acc.cgi?acc=GSE8894) | [19010856](https://www.ncbi.nlm.nih.gov/pubmed/19010856) | GEO (Lee) | Affymetrix HG-U133_Plus_2 | 75 |
| [GSE16534](https://www.ncbi.nlm.nih.gov/geo/query/acc.cgi?acc=GSE16534) | [19737969](https://www.ncbi.nlm.nih.gov/pubmed/19737969) | GEO (Lin) | Affymetrix HG 1.0 ST Exon Array | 22 |
| [GSE28571](https://www.ncbi.nlm.nih.gov/geo/query/acc.cgi?acc=GSE28571) | [22011649](https://www.ncbi.nlm.nih.gov/pubmed/22011649) | GEO (Micke) | Affymetrix HG-U133_Plus_2 | 28 |
| [GSE74777](https://www.ncbi.nlm.nih.gov/geo/query/acc.cgi?acc=GSE74777) | [27613525](https://www.ncbi.nlm.nih.gov/pubmed/27613525) | GEO (Noro R) | Affymetrix Human Transcriptome Array 2.0 | 107 |
| [GSE4573](https://www.ncbi.nlm.nih.gov/geo/query/acc.cgi?acc=GSE4573) | [16885343](https://www.ncbi.nlm.nih.gov/pubmed/16885343) | GEO (Raponi) | Affymetrix HG-U133A | 130 |
| [GSE30219](https://www.ncbi.nlm.nih.gov/geo/query/acc.cgi?acc=GSE30219) | [23698379](https://www.ncbi.nlm.nih.gov/pubmed/23698379) | GEO (Rousseaux) | Affymetrix HG-U133_Plus_2 | 96 |
| [GSE18842](https://www.ncbi.nlm.nih.gov/geo/query/acc.cgi?acc=GSE18842) | [20878980](https://www.ncbi.nlm.nih.gov/pubmed/20878980) | GEO (Sánchez-Palencia) | Affymetrix HG-U133_Plus_2 | 31 |
| [GSE41271](https://www.ncbi.nlm.nih.gov/geo/query/acc.cgi?acc=GSE41271) | [23449933](https://www.ncbi.nlm.nih.gov/pubmed/23449933) | GEO (Sato) | Illumina HumanWG-6 v3.0 | 80 |
| [GSE29016](https://www.ncbi.nlm.nih.gov/geo/query/acc.cgi?acc=GSE29016) | [22676229](https://www.ncbi.nlm.nih.gov/pubmed/22676229) | GEO (Staaf) | Illumina HumanHT-12 v3.0 | 13 |
| [GSE42127](https://www.ncbi.nlm.nih.gov/geo/query/acc.cgi?acc=GSE42127) | [23357979](https://www.ncbi.nlm.nih.gov/pubmed/23357979) | GEO (Tang) | Illumina HumanWG-6 v3.0 | 43 |
| [TCGA-LUSC](https://www.ncbi.nlm.nih.gov/geo/query/acc.cgi?acc=GSE72094) | [22960745](http://www.ncbi.nlm.nih.gov/pubmed/22960745) | cBioPortal | RNA-Seq (Illumina HiSeq 2000) | 501 |
| [GSE23822](https://www.ncbi.nlm.nih.gov/geo/query/acc.cgi?acc=GSE23822) | [22514692](https://www.ncbi.nlm.nih.gov/pubmed/22514692) | GEO (Wright) | Illumina HumanHT-12 v3.0 | 56 |
| [GSE14814](https://www.ncbi.nlm.nih.gov/geo/query/acc.cgi?acc=GSE14814) | [20823422](https://www.ncbi.nlm.nih.gov/pubmed/20823422) | GEO (Zhu) | Affymetrix HG-U133A | 52 |
| **Total** |  |  |  | **2088** |

**Table S2.** Summary of clinicopathological variables by SCC consensus subtype.

|  |  | SCC1 | SCC2 | SCC3 | SCC4 | SCC5 |  |
| --- | --- | --- | --- | --- | --- | --- | --- |
|  | N | N = 207 | N = 499 | N = 538 | N = 646 | N = 196 | *p* value |
| **Sex, N (%)** | **1949** |  |  |  |  |  | **0.097** |
| Male |  | 165 (83.33) | 350 (75.27) | 391 (78.04) | 486 (81.00) | 145 (78.38) |  |
| Female |  | 33 (16.67) | 115 (24.73) | 110 (21.96) | 114 (19.00) | 40 (21.62) |  |
| **Age, N (%)** | **1808** |  |  |  |  |  | **0.252** |
| ≤ 50 |  | 11 (5.85) | 30 (6.96) | 28 (6.15) | 31 (5.51) | 13 (7.60) |  |
| > 50 and ≤ 65 |  | 83 (44.15) | 159 (36.89) | 207 (45.49) | 252 (44.76) | 78 (45.61) |  |
| > 65 |  | 94 (50.00) | 242 (56.15) | 220 (48.35) | 280 (49.73) | 80 (46.78) |  |
| **Stage, N (%)** | **1561** |  |  |  |  |  | **0.324** |
| Early-Stage (I - II) |  | 133 (84.18) | 330 (86.16) | 317 (83.20) | 407 (81.24) | 119 (86.23) |  |
| Late-Stage (III - IV) |  | 25 (15.82) | 53 (13.84) | 64 (16.80) | 94 (18.76) | 19 (13.77) |  |
| **Smoking history, N (%)** | **1161** |  |  |  |  |  | **0.099** |
| Never-smoker |  | 7 (5.98) | 11 (3.96) | 14 (4.75) | 8 (2.18) | 1 (0.96) |  |
| Smoker |  | 110 (94.02) | 267 (96.04) | 281 (95.25) | 359 (97.82) | 103 (99.04) |  |

**Table S3.** FDR adjusted *p* values for pairwise comparisons of TMB values between SCC subtypes.

|  | **SCC1** | **SCC2** | **SCC3** | **SCC4** |
| --- | --- | --- | --- | --- |
| **SCC2** | 0.036 | - | - | - |
| **SCC3** | 0.89 | 0.025 | - | - |
| **SCC4** | 0.89 | 0.013 | 0.89 | - |
| **SCC5** | 0.89 | 0.124 | 0.89 | 0.89 |

**Table S4.** FDR adjusted *p* values for pairwise comparisons of copy number alterations rate between SCC subtypes.

|  | **SCC1** | **SCC2** | **SCC3** | **SCC4** | |
| --- | --- | --- | --- | --- | --- |
| **SCC2** | 8.1e-10 | - | - | - |  |
| **SCC3** | 0.00058 | 2.5e-10 | - | - |  |
| **SCC4** | 0.31 | 5.2e-13 | 0.0016 | - |  |
| **SCC5** | 0.014 | 0.015 | 0.85 | 0.053 |  |

**Table S5.** FDR adjusted *p* values for pairwise comparisons of DNA damage repair (DDR) deficiency score between SCC subtypes.

|  | **SCC1** | **SCC2** | **SCC3** | **SCC4** |
| --- | --- | --- | --- | --- |
| **SCC2** | 0.0015 | - | - | - |
| **SCC3** | 0.029 | 0.074 | - | - |
| **SCC4** | 0.025 | 0.074 | 0.91 | - |
| **SCC5** | 0.0082 | 0.91 | 0.14 | 0.2 |

**Table S6.** Subtype specific gene expression signatures. Upregulated and downregulated genes in each subtype versus the other subtypes based on differential gene expression analysis on the biggest dataset of the discovery cohort, TCGA-LUSC (n > 500).

| **Lung SCC subtype** | **Upregulated genes** | **Downregulated genes** |
| --- | --- | --- |
| SCC1 | *AK4, ASPH, CCT6A, EIF4EBP1, ERLIN2, FAM83H, FKBP9P1, FOSL1, FSCN1, LPAR3, MAGEA6, MRPS17, MYC, PGF, PTHLH, PUS7, SH2D5, SLC3A2, STC2, STEAP1, TFRC, TYRO3* | *ACKR1, AMT, AMY2B, C2orf40, CD1E, CLDN11, CYFIP2, CYP3A5, FRZB, HID1, IL34, INMT, LIME1, MAP4K1, MUC15, N4BP2L1, NDRG2, PLA2G2A, PLEKHB1, PTPRN2, RAB37, RBP5, RHOU, SLAIN1, XCL2* |
| SCC2 | *A2M, ABCA3, ABCA6, ABCA8, ABCC6, ABCC9, ABI3BP, ABLIM3, ACAA2, ACE, ACKR1, ACOX2, ACP5, ACSM3, ACSS3, ACTA2, ACTG2, ACTN1, ACVRL1, ADAM12, ADAM19, ADAM6, ADAM8, ADAMTS10, ADAMTS12, ADAMTS16, ADAMTS2, ADAMTS7, ADAMTS8, ADAMTS9, ADAMTSL2, ADAMTSL4, ADARB1, ADCY4, ADGRA2, ADGRD1, ADGRE2, ADGRE5, ADGRF1, ADGRF5, ADGRL4, ADH1B, ADORA1, ADORA2A, ADRA2A, ADTRP, AEBP1, AGER, AGPAT4, AGR3, AGT, AGTR2, AHCYL2, AKAP12, AKAP2, ALDH1A2, ALDH1A3, ALDH2, ALDH3B1, ALOX15B, ALOX5, ALPK2, ALPK3, ALPL, AMIGO2, AMY1A, ANG, ANGPT1, ANGPTL2, ANK2, ANKRD1, ANKRD36BP2, ANOS1, ANPEP, ANTXR1, ANTXR2, ANXA3, ANXA6, AOC3, AOX1, APBB1IP, APBB2, APCDD1, APLNR, APOBR, APOC2, APOD, AQP1, AQP3, AQP4, AREG, ARHGAP15, ARHGAP29, ARHGAP31, ARHGEF10L, ARHGEF15, ARHGEF17, ARHGEF40, ARHGEF6, ARRB1, ASAH1, ASPN, ATOH8, ATP10A, ATP11A, ATP13A4, ATP2A3, ATP6V0D2, ATP8B2, ATP8B4, AXIN2, AXL, AZGP1, B3GNT7, B3GNT8, BANK1, BCAS1, BCL6B, BGN, BHLHE22, BHLHE41, BICC1, BLK, BMF, BMP2, BMP5, BMS1P20, BNC2, BOC, BST1, BTD, BTG2, BTK, C10orf10, C10orf54, C11orf96, C14orf132, C16orf45, C16orf54, C16orf89, C19orf38, C1orf116, C1orf54, C1QTNF1, C1QTNF3, C1QTNF7, C1R, C20orf85, C2orf40, C2orf54, C3, C4A, C4BPA, C5AR2, C5orf49, C7, C8orf4, CA3, CACNA1C, CACNA2D2, CADM1, CADM3, CADPS2, CALD1, CALHM2, CAMK2N1, CAPG, CAPN13, CAPN8, CAPS, CAPSL, CARD6, CARD9, CASS4, CAV1, CCDC102B, CCDC170, CCDC69, CCDC80, CCDC92, CCL14, CCL21, CCL22, CCL7, CCNJL, CCPG1, CCR4, CCR7, CD180, CD19, CD200, CD22, CD248, CD27, CD28, CD300E, CD300LB, CD302, CD34, CD36, CD37, CD5, CD52, CD55, CD59, CD63, CD79A, CD79B, CD93, CDC42EP5, CDH11, CDH23, CDH5, CDH6, CEACAM1, CEACAM21, CEACAM4, CEACAM6, CECR1, CELF2, CEMIP, CFAP221, CFH, CFI, CFLAR, CFP, CFTR, CGNL1, CH25H, CHI3L1, CHIA, CHN1, CHRDL1, CHRDL2, CHST11, CILP, CILP2, CISH, CITED2, CLDN18, CLDN2, CLDN23, CLDN5, CLEC10A, CLEC11A, CLEC14A, CLEC3B, CLEC5A, CLIC3, CLIC5, CLIC6, CLIP3, CLMP, CMAHP, CMTM3, CNN1, CNN3, CNRIP1, COL10A1, COL11A1, COL12A1, COL14A1, COL15A1, COL16A1, COL1A1, COL1A2, COL3A1, COL4A1, COL4A2, COL4A3, COL4A4, COL5A1, COL5A2, COL6A1, COL6A2, COL6A3, COL6A5, COL6A6, COL8A1, COL8A2, COLEC12, COMP, CORIN, COTL1, COX4I2, COX7A1, CPA3, CPA4, CPAMD8, CPB2, CPED1, CPNE5, CPQ, CPXM1, CPZ, CREB3L1, CREB3L2, CRIP1, CRIP2, CRISPLD2, CRTAC1, CRYM, CSDC2, CSF1, CSF3R, CSGALNACT1, CSRNP1, CST2, CST6, CTGF, CTHRC1, CTSB, CTSD, CTSE, CTSG, CTSH, CTSK, CTSO, CTSZ, CXCL12, CXCL16, CXCL17, CXCL2, CXCR2, CXorf36, CXXC5, CYB5R1, CYBA, CYBRD1, CYP27A1, CYP2B7P, CYP39A1, CYP4B1, CYP7B1, CYR61, CYSLTR1, CYTH4, CYYR1, DAAM2, DAB2, DACT1, DACT3, DAPK1, DCHS1, DCN, DCSTAMP, DDAH1, DDR2, DEFB4A, DENND2A, DERL3, DES, DHRS9, DKK2, DLC1, DLG2, DLL4, DMBT1, DNAAF1, DNALI1, DNM3, DOCK10, DOCK11, DOCK2, DOCK4, DOCK8, DOK3, DOK5, DPEP1, DPEP2, DPP4, DPT, DPYD, DPYSL2, DPYSL3, DTX4, DUSP1, DUSP5, DYNLRB2, DYSF, ECM1, ECM2, ECSCR, EDIL3, EDNRA, EDNRB, EFEMP1, EFEMP2, EGFL6, EGR3, EHD2, ELK3, ELN, EMB, EMCN, EMILIN1, EMILIN2, EMP3, ENC1, ENG, ENPP2, ENTPD1, EPAS1, EPS8L1, EPYC, ERG, ERP27, ERRFI1, ESAM, ETHE1, ETS1, ETV1, EVA1A, F10, F13A1, F2RL3, F3, F5, FABP3, FABP4, FAM101A, FAM101B, FAM107A, FAM107B, FAM13C, FAM180A, FAM189A2, FAM198B, FAM20A, FAM20C, FAM26E, FAM46B, FAM46C, FAM49A, FAM65B, FAM78A, FAM92B, FAP, FAT4, FAXDC2, FBLN1, FBLN2, FBLN5, FBN1, FBP1, FBXL7, FBXO2, FBXO32, FCER1A, FCGR3B, FCGRT, FCMR, FCRL2, FCRL5, FCRLA, FER1L4, FERMT2, FGA, FGD2, FGD5, FGF14, FGF7, FGG, FGR, FHAD1, FHL1, FHL5, FIBIN, FILIP1L, FKBP11, FKBP7, FLI1, FLNA, FLRT3, FLT4, FMNL1, FMO1, FMO2, FMO3, FMO5, FMOD, FN1, FNDC1, FNIP2, FOLR1, FOSB, FOXA2, FOXF1, FOXP1, FRMD4A, FRY, FRZB, FSTL1, FSTL3, FUCA1, FXYD1, FZD4, G0S2, GAB2, GADD45B, GALC, GALM, GALNT10, GALNT15, GALNT5, GAS1, GAS6, GAS7, GATA6, GBP3, GCOM1, GDPD5, GEM, GFPT2, GGT5, GGTA1P, GGTLC1, GIMAP1, GIMAP8, GJA4, GJA5, GJD3, GKN2, GLI3, GLIPR1, GLIS2, GLT8D2, GNA14, GNG11, GPC6, GPIHBP1, GPR132, GPR171, GPR39, GPR68, GPRC5A, GPRIN3, GPSM3, GPX3, GRAMD3, GRAP2, GRASP, GREM1, GRK5, GSTM5, GUCY1A3, GXYLT2, GYPC, GZMK, HABP2, HAMP, HAPLN3, HAS2, HBA2, HBB, HDC, HEG1, HEPH, HERPUD1, HEYL, HGF, HHIP, HIC1, HIGD1B, HLA-DOB, HLA-DQA2, HLA-DQB1, HLA-G, HMCN1, HNF1B, HNMT, HOPX, HOXB2, HP, HPGD, HPGDS, HS3ST2, HSD17B6, HSPA12B, HSPB2, HSPB6, HSPB7, HSPB8, HSPG2, HTRA1, HTRA3, HYAL1, ICAM3, ICAM4, IFFO1, IFITM2, IFNAR2, IGF1, IGFBP4, IGFBP7, IGFL2, IKZF1, IL16, IL1R1, IL1RL1, IL33, IL34, IL3RA, IL4R, IL6ST, IL7R, INHBA, INMT, INPP5D, IQGAP2, IQSEC1, IRAK3, IRF4, IRF7, ISLR, ISM1, ITGA1, ITGA11, ITGA3, ITGA4, ITGA5, ITGA8, ITGA9, ITGAL, ITGAX, ITGB3, ITGB6, ITGBL1, ITIH5, ITM2A, ITPR2, ITPRIP, JAM2, JAM3, JAML, JSRP1, KANK2, KANK4, KCNA3, KCNAB2, KCNE4, KCNJ5, KCNJ8, KCNK3, KCNK6, KCNMB1, KCNN3, KCNN4, KCNQ1, KCTD12, KCTD14, KDR, KIAA0040, KIAA0125, KIAA0513, KIAA1462, KIF13B, KIF26B, KIRREL, KIT, KLF10, KLF2, KLF6, KLF9, KLHDC7B, KLHL6, KLK5, KRT7, LAMA2, LAMA4, LAMB2, LAMC2, LAMP3, LAMP5, LATS2, LAX1, LCP1, LDB2, LDLRAD4, LGALS1, LGI2, LGMN, LHFP, LHFPL2, LIF, LIMCH1, LIMS2, LINC00341, LINC01140, LIPG, LMCD1, LMO2, LMO3, LMOD1, LOC100129034, LOX, LOXL1, LOXL2, LOXL4, LPIN2, LPL, LRMP, LRP1, LRRC15, LRRC17, LRRC32, LRRC6, LRRK2, LRRN1, LRRN4, LSP1, LTBP2, LTC4S, LTF, LUM, LXN, LY9, LYVE1, LZTS1, MACROD2, MAFB, MAL, MALL, MAMDC2, MAN1A1, MAN1C1, MAOB, MAP3K8, MARCO, MATN3, MCAM, MCEMP1, ME3, MEDAG, MEF2B, MEF2C, MEG3, MEI1, MEOX2, METTL7A, MFAP2, MFAP4, MFGE8, MFNG, MFRP, MFSD7, MGLL, MGP, MICAL2, MIR100HG, MIR22HG, MITF, MLPH, MMP13, MMP14, MMP19, MMP2, MMP23B, MMP28, MMP7, MMP9, MMRN1, MMRN2, MOCS1, MPP1, MRAS, MRC2, MRGPRF, MRVI1, MS4A2, MS4A8, MSLN, MSRB3, MT1E, MT1L, MT1M, MT2A, MUC1, MUC21, MUC5B, MVP, MXRA5, MXRA8, MYADM, MYCT1, MYL9, MYLK, MYO1F, MYO1G, MZB1, NAAA, NAPSA, NAPSB, NBL1, NCALD, NCAM2, NCCRP1, NCF1B, NCF4, NDN, NDNF, NEDD9, NETO1, NEXN, NFATC2, NID2, NINJ2, NKX2-1, NLRC4, NLRP3, NME5, NNMT, NOSTRIN, NOTCH4, NOX4, NPC2, NPR1, NR2F1, NR4A3, NRGN, NRP1, NRP2, NTM, NTN4, NUPR1, OGN, OLFML1, OLFML2B, OLFML3, OMD, OPN3, ORM1, ORM2, P2RX1, P2RX7, P2RY6, P2RY8, P4HA3, PADI2, PAEP, PALM2-AKAP2, PALMD, PAMR1, PAPSS2, PARM1, PARVG, PCDH1, PCDH12, PCDH17, PCDHGA12, PCOLCE, PCP4, PCSK5, PDE1A, PDGFC, PDGFD, PDGFRA, PDGFRB, PDGFRL, PDK4, PDLIM2, PDLIM3, PDPN, PDZD2, PDZK1IP1, PDZRN3, PEAR1, PEBP4, PECAM1, PENK, PER1, PGC, PGM5, PHLDB1, PID1, PIEZO2, PIFO, PIGR, PIK3CG, PIK3IP1, PIK3R5, PIM2, PINK1, PIP, PKNOX2, PLA1A, PLA2G1B, PLA2G2A, PLA2G4E, PLA2G5, PLAUR, PLCG2, PLCL2, PLD4, PLEKHA4, PLEKHO2, PLEKHS1, PLIN2, PLK3, PLN, PLPP4, PLVAP, PLXDC1, PLXDC2, PLXND1, PMEPA1, PMP22, PNMA2, PNOC, PODN, PODNL1, PODXL, POSTN, POU2AF1, POU2F2, PPM1K, PPP1R12B, PPP1R14A, PPP1R16B, PRAM1, PRDM1, PRDM6, PRELP, PREX1, PRG4, PRICKLE1, PRICKLE2, PRKCDBP, PRKG1, PROS1, PRRX1, PRSS23, PSAP, PTGDS, PTGER2, PTGIR, PTGIS, PTGS1, PTGS2, PTH1R, PTP4A3, PTPN7, PTPRB, PTPRE, PTPRM, PTPRN2, PTRF, PXDC1, PXDN, QPRT, RAB20, RAB31, RAB8B, RAI2, RAMP1, RAMP2, RAMP3, RAPGEF3, RARRES1, RARRES2, RASA3, RASGEF1B, RASGRF1, RASGRF2, RASGRP2, RASGRP3, RASGRP4, RASIP1, RASL12, RASSF2, RASSF4, RBMS2, RBP4, RBPMS, RCAN2, RCN3, RETN, RFTN1, RGCC, RGN, RGS16, RGS2, RGS3, RGS4, RGS5, RHOBTB2, RHOJ, RIN3, RNASE4, RND1, ROBO4, ROR1, RORC, ROS1, RPS6KA2, RRAD, RRAS, RSPH1, RSPH9, RSPO3, RUNX2, S100A4, S1PR1, S1PR4, SAMD11, SAMD4A, SCARF1, SCARF2, SCEL, SCGB3A1, SCGB3A2, SCN1B, SCN7A, SCNN1B, SCNN1G, SCT, SCTR, SDC3, SDC4, SDR16C5, SELENBP1, SELL, SELP, SEMA3B, SEMA3C, SEMA5A, SEMA6B, SEPP1, SEPT4, SERPINA3, SERPINB7, SERPIND1, SERPINE1, SERPINF1, SERTAD1, SFRP2, SFRP4, SFTA1P, SFTA2, SFTA3, SFTPA1, SFTPA2, SFTPB, SFTPC, SFTPD, SFXN3, SGCA, SGCD, SGMS2, SH2B3, SH2D3C, SH3BGRL, SH3BP5, SH3PXD2B, SH3TC1, SHE, SHROOM4, SIDT1, SIGLEC12, SIGLEC5, SIRPA, SIRPB1, SIRPB2, SLAMF1, SLAMF6, SLC11A1, SLC16A2, SLC16A4, SLC1A1, SLC22A3, SLC24A3, SLC26A9, SLC2A10, SLC2A5, SLC34A2, SLC38A5, SLC40A1, SLC43A3, SLC44A4, SLC4A4, SLC5A8, SLC6A14, SLC7A2, SLC9A3R2, SLCO2A1, SLFN11, SLIT2, SLIT3, SLPI, SMAD7, SMIM10, SMIM3, SMPD1, SMPDL3A, SMPDL3B, SNAI1, SNED1, SNRPN, SNTB1, SOD3, SORBS1, SORBS2, SORCS2, SOX17, SP110, SP140, SPAG4, SPARC, SPATA13, SPATA18, SPDEF, SPINK1, SPNS2, SPOCD1, SPOCK2, SPON1, SPON2, SPRY1, SPTLC3, SRPX2, SSC5D, SSPN, ST3GAL5, ST5, ST6GAL1, ST6GALNAC5, ST6GALNAC6, STAB1, STARD13, STARD8, STEAP4, STK10, STRA6, STS, STX11, SULF1, SULT1C2, SULT1C4, SUSD2, SVEP1, SYNC, SYNE1, SYNPO, SYNPO2, SYT15, SYTL2, SYTL3, TAGLN, TARP, TBX4, TBX5, TCEAL7, TCF21, TEK, TESC, TFPI, TGFB1I1, TGFB2, TGFB3, TGFBI, TGFBR2, THBS1, THBS2, THY1, TIE1, TIMP1, TIMP2, TIMP3, TJP3, TLR2, TLR7, TM4SF18, TM6SF1, TMC5, TMEM100, TMEM106A, TMEM119, TMEM125, TMEM130, TMEM156, TMEM173, TMEM176A, TMEM176B, TMEM190, TMEM200A, TMEM204, TMEM255B, TMEM45A, TMEM47, TMEM51, TMEM86A, TMPRSS2, TNC, TNFAIP3, TNFAIP6, TNFRSF10C, TNFRSF12A, TNFRSF13B, TNFRSF17, TNFRSF19, TNFSF13, TNFSF8, TNNC1, TNS1, TNS2, TNS3, TNXB, TOR4A, TP53INP1, TPK1, TPM1, TPM2, TPP1, TPPP3, TPSAB1, TPSB2, TRAM1, TREM1, TRIM34, TRPC6, TRPV2, TSC22D3, TSHZ3, TSPAN1, TSPAN2, TSPAN4, TSPAN8, TUBA1A, TXNIP, UBA7, UBE2QL1, UBXN10, UNC13D, UNC5B, UNC5C, VASH1, VASN, VCAN, VDR, VEGFC, VEPH1, VGLL3, VILL, VIM, VPREB3, VSIG2, VSTM2L, VSTM4, VWA2, VWA5A, VWF, WAS, WBP1L, WDFY4, WFDC1, WFDC2, WIPF1, WISP1, WISP2, WNT10A, WNT2, WNT4, WTIP, WWC2, XBP1, XYLT1, ZBP1, ZBTB47, ZC4H2, ZCCHC24, ZEB1, ZEB2, ZFP36, ZFPM2, ZG16B, ZMYND10, ZNF385B, ZNF423, ZNF521* | *ABCC5, ABCF3, ACTL6A, ACTR3B, ACYP1, ADCY10, ADRA2B, AHCY, AK4, AKR1C1, AKR1C3, ALG3, ALOX12P2, ANKRD13B, APTX, ARHGEF39, ARTN, ASF1B, ASNS, ASPM, ATAD2, ATG4D, ATP5G1, ATP6V1E2, AUNIP, AURKA, AURKB, B4GALNT1, BDH1, BLM, BNIP3, BOLA3, BORA, BRCA1, BRI3BP, BTBD11, BUB1B, C11orf84, C12orf56, C12orf73, C16orf59, C17orf53, C17orf75, C17orf89, C19orf48, C1orf112, C1QBP, C3orf67, CABYR, CACNA1B, CACYBP, CBS, CBX2, CCDC138, CCDC58, CCDC59, CCNB1IP1, CCNE1, CCNE2, CCT4, CDC25A, CDC25C, CDCA2, CDCA3, CDCA7, CDCA8, CDH24, CDK1, CDT1, CENPA, CENPH, CENPI, CENPK, CENPM, CENPN, CENPO, CENPQ, CENPV, CENPW, CGREF1, CHAF1B, CHRNA5, CHTF18, CKMT1A, CKMT1B, CMAS, CMSS1, CNNM1, COA6, COLEC11, CT45A5, CYC1, DANCR, DARS2, DBF4, DBF4B, DCTPP1, DDTL, DDX11, DDX12P, DDX39A, DEPDC1B, DGKG, DKC1, DLEU2, DLX6, DNA2, DNAH14, DNAJC19, DNAJC9, DNMT3B, DSCC1, E2F1, ECE2, ECT2, EME1, EMG1, EPCAM, ERCC6L, ESPL1, ETV4, EXOSC3, EZH2, F12, FAM122B, FAM162A, FAM195A, FAM201A, FAM60A, FAM72B, FAM72D, FAM83D, FANCE, FANCG, FANCI, FANCL, FBL, FEN1, FKBP4, FOXM1, FXR1, FZD9, G6PD, GAL, GCLC, GCLM, GCSH, GINS1, GINS2, GINS3, GINS4, GJB7, GLS2, GMPS, GPX2, GSG2, GSR, GTPBP3, GTSE1, H2AFX, HAP1, HAUS8, HELLS, HES6, HIST3H2A, HJURP, HMMR, HOXA10, HOXB7, HPDL, HPRT1, HS6ST2, HSPE1, IQGAP3, JAKMIP3, KAT2A, KCNMB3, KIAA0101, KIAA1524, KIF11, KIF15, KIF18B, KIF20A, KIF23, KIF2C, KIF4A, KIFC1, KLRG2, KNSTRN, KRTCAP3, LDHB, LIN9, LMNB1, LOC220729, LOC642846, LOC728554, LOC730101, LRP8, LSM4, MAP6D1, MCM10, MCM2, MCM4, MCM7, ME1, MED30, MELTF, MFN1, MND1, MRPL12, MRPL21, MRPL47, MRPS23, MRPS26, MTBP, MTL5, MZT1, MZT2A, MZT2B, NCBP2-AS2, NDC80, NDUFB5, NDUFB9, NECAB2, NEK2, NME2, NPM3, NQO1, NR2C2AP, NRARP, NSUN5, NT5M, NUDT1, NUDT8, NUF2, NUP37, NUP62CL, NUSAP1, ODC1, OIP5, OLA1, ORC6, PAICS, PAM16, PANX2, PARL, PCCB, PCNA, PDCD10, PDCD2L, PDSS1, PGD, PHGDH, PIF1, PIGX, PIR, PKMYT1, PNO1, PNPT1, POC1A, POLE, POLE2, POLR2H, PPAN, PRAME, PRC1, PRR7, PSAT1, PSMC3IP, PTTG1, RAC3, RACGAP1, RAD51, RAD51AP1, RAD54B, RAD54L, RCCD1, RECQL4, RFC2, RFC3, RFC4, RFC5, RMI1, RMI2, RNASEH2A, RPA3, RPL22L1, RPL35A, RPL36A, RPL39L, RPS7, SAPCD2, SCARNA12, SDHAF3, SGOL2, SHFM1, SIGMAR1, SKA1, SKA3, SKP2, SLC25A5, SLC26A10, SLC35G1, SLC47A2, SLC7A11, SMC4, SNHG1, SNHG17, SNORA50C, SNRPD1, SNRPE, SNRPF, SNRPG, SOX2, SPAG5, SRXN1, STIL, STOML2, STRA13, SURF2, SUV39H1, TACC3, TALDO1, TBL1X, TFAP4, TFR2, TFRC, THAP7-AS1, THOC3, TIMELESS, TIPIN, TK1, TKT, TMEM161A, TMEM246, TMEM38B, TMEM52, TMEM97, TOMM5, TONSL, TOP1MT, TOP2A, TPRXL, TRAIP, TRIB3, TRIM16L, TRIP13, TROAP, TXN, TXNRD1, TYMSOS, TYSND1, UBE2C, UBE2S, UCHL1, UCK2, UGT1A9, UGT8, UNG, UPF3B, VRK1, WBSCR27, WNK2, XRCC2, XXYLT1, YDJC, YEATS4, ZC3H8, ZMYND19, ZNF367, ZNF639, ZNF695, ZP3, ZWINT* |
| SCC3 | *AIM2, ANKRD22, APOBEC3G, ASS1, BCL2L14, CASP4, CCL8, CD274, CD7, CMPK2, CRTAM, CXCR2P1, DDX60, ETV7, FASLG, FBXO6, GBP1P1, GNLY, GZMB, HCP5, HERC5, HERC6, IFI27, IFI35, IFI6, IFIT1, IFIT2, IFITM1, IFNG, ISG15, KLRC1, KLRC2, KLRD1, KYNU, LAG3, LGALS9, MX1, NMI, NR1H3, OAS1, OAS3, PARP9, PSMB10, PSME1, PSME2, RAB27B, SERPINB9, SNX10, STAT1, TAP1, TRIM21, UBD, WARS, ZNF683* | *ADRA2C, ARVCF, BAIAP2-AS1, BEX4, BRD1, BRF1, C20orf194, C22orf29, CACHD1, CADM4, CDH26, CEP250, CLTCL1, COL7A1, DGCR2, DGCR6, DST, DUOX1, DVL3, EFS, EMILIN3, EPHA4, FBXL19, FRAS1, GABBR1, IGFBP2, IPW, JMJD7-PLA2G4B, KCTD15, KIF7, KIFC2, KLF8, KRBA1, LAMA5, LOC100132287, MAGED4, MAPK8IP3, MICAL3, MTA1, NEAT1, NSG1, NUPR2, NYNRIN, PI4KAP1, PIANP, PITPNM3, PKD1, PLA2G6, PLXNB1, PRODH, RAB11FIP3, SDK1, SDK2, SEPT5, SIPA1L2, SLC22A23, SLC4A3, SMTN, SORL1, SPEG, STON2, SYNGR1, TCEAL2, TENM4, TMEM178A, TNK2, TRO, TTC7B, TTLL3, TUBGCP6, TUG1, VIPR1, WLS, ZDHHC8, ZNF117, ZNF589, ZNF74* |
| SCC4 | *ACSS1, AIF1L, ALDH1A1, ARHGEF39, ATAD5, ATP6V1B1, ATP6V1C2, BCL11A, BEX2, BLM, BLMH, BRCA1, BUB1B, C16orf59, C17orf96, C1orf226, C2orf15, C3orf58, CABYR, CAMSAP3, CBS, CBX1, CCDC14, CENPF, CHAF1A, CHST10, COCH, DHRS11, DHX33, E2F7, ELOVL6, EME1, ENHO, EPCAM, EPHA7, ERICH5, ESRG, EZH2, FOXRED2, GCLC, GKAP1, GPT2, GRHL1, GSTA1, GSTA4, GSTM3, GTSE1, ICK, IGSF9, KCNJ11, KIAA1324, KIAA1804, KIF18B, KIF21A, KLF5, KLHL23, KLRG2, KRT8, LIG3, LMNB2, LOC100133985, LOC730101, MARC1, MCCC1, MCM3, MCM8, MSH6, MYB, MYEF2, MYLIP, NEB, NMNAT3, OTX1, OXGR1, PABPC1L, PART1, PAX6, PHLPP1, POF1B, PPM1H, PRKX, PSMC3IP, RAB3B, RAD51C, RAD54L, RAPGEFL1, RFC4, RIPPLY3, RMI2, RUSC1-AS1, SAPCD2, SBK1, SCML2, SEMA6C, SHMT1, SLC25A10, SLC7A11, SLC9A4, SOX21, SPAG5, STK26, STMN1, STRBP, STXBP6, SUZ12P1, TADA2A, TICRR, TMEM44, TMEM97, TTLL12, UGT8, USH1C, USP13, VSIG10, WASF1, WDR62, WNK2, ZNF711* | *ACAP1, ACTA2, ADAM12, ADAM19, ADAM28, ADAM8, ADAMTS12, ADAMTS2, ADAMTS4, ADGRE5, ADRB2, AEBP1, AFAP1L2, AIM2, ALDH1A3, ALOX15B, ALOX5, ALOX5AP, AMIGO2, ANGPTL2, ANGPTL4, ANPEP, ANTXR2, ANXA6, ANXA8, APCDD1L, AQP9, ARHGAP15, ARHGAP9, ARHGEF6, ARL14, ARSI, ASPN, AXL, BASP1, BCAR3, BCL2A1, BGN, BHLHE41, BICC1, BMP1, BMP2, BTK, C10orf10, C10orf54, C11orf96, C15orf48, C1QTNF1, C1R, C1S, C3, C5orf46, CALD1, CAMK2N1, CAV1, CCDC69, CCL2, CCL20, CCL22, CCL3, CCL3L1, CCL4L1, CCR6, CD14, CD19, CD1A, CD22, CD248, CD37, CD68, CD69, CD74, CD79B, CDC42EP5, CDH11, CDK14, CFB, CFH, CHN1, CHST11, CHST15, CIITA, CILP, CLEC11A, CLEC2B, CLEC5A, CLMP, CNN1, CNRIP1, COL10A1, COL11A1, COL12A1, COL15A1, COL16A1, COL1A1, COL1A2, COL3A1, COL5A1, COL5A2, COL5A3, COL6A1, COL6A2, COL6A3, COL8A1, COL8A2, COLEC12, COMP, CORO1A, CPXM1, CPZ, CREB3L1, CRIP1, CRIP2, CRISPLD2, CSF1R, CSF2, CSF3, CST7, CTGF, CTHRC1, CTSB, CTSH, CTSK, CXCL1, CXCL12, CXCL13, CXCL2, CXCL6, CXXC5, CYR61, CYTH4, CYTIP, DAB2, DACT1, DCN, DDIT4, DENND3, DERL3, DIO2, DOK3, DPEP2, DPT, DPYD, DUSP1, DUSP6, ECM2, EDNRA, EFEMP2, EHD2, EMILIN1, EMP3, ENC1, EPSTI1, EREG, ETS1, EVA1A, FAM101A, FAM107B, FAM198B, FAM20A, FAM20C, FAP, FAS, FBLN2, FBLN5, FBN1, FBXO2, FCER1A, FCGR2A, FCGR2B, FCMR, FCRLA, FGD2, FHL2, FIBIN, FILIP1L, FLT3LG, FMNL1, FMOD, FN1, FNDC1, FPR1, FPR2, FSTL3, FXYD5, FYB, G0S2, GAS1, GAS7, GATA3, GEM, GFPT2, GGT5, GGTA1P, GLIPR1, GLT8D2, GMFG, GNG2, GPC6, GPR132, GPR68, GPSM3, GREM1, GXYLT2, GYPC, HAS2, HCST, HEPH, HIVEP3, HLA-DMA, HLA-DPB1, HLA-DQB2, HRH1, HTRA1, HTRA3, ICAM1, ICAM3, IFFO1, IFITM1, IFITM2, IGFBP7, IGFL2, IL10RA, IL15RA, IL16, IL1A, IL1B, IL32, IL33, IL4R, IL6, IL7R, INHBA, INPP1, INPP4B, IRAK2, ISLR, ITGA1, ITGA11, ITGA3, ITGA4, ITGA5, ITGAV, ITGAX, ITGB6, JAK3, JAML, KIFC3, KLHL6, LAMA4, LAMC2, LAT2, LCP1, LCP2, LEPROT, LGALS1, LINC00152, LIPG, LOC606724, LOXL1, LOXL2, LRRC15, LRRC17, LST1, LTB, LTBP2, LUM, LY75, LYPD1, MCEMP1, MCTP1, MEDAG, MEF2C, MEI1, MFAP4, MICAL2, MIR100HG, MIR31HG, MMP1, MMP12, MMP13, MMP2, MMP28, MMP3, MMP7, MMP9, MRAS, MRC2, MRGPRF, MS4A1, MSN, MT2A, MVP, MXRA5, MXRA8, MYADM, MYL9, MYO1G, NAPSB, NBL1, NCF4, NCR3, NEXN, NID2, NNMT, NRP1, NRP2, ODF3B, OLFML1, OLFML2B, OLFML3, OMD, OPTN, OSM, OSMR, P2RY8, P4HA3, PALMD, PARVG, PCED1B, PCOLCE, PDGFRA, PDGFRB, PDGFRL, PDLIM3, PDLIM7, PDPN, PDZRN3, PHLDA2, PLAT, PLAUR, PLD4, PLEK2, PLEKHF1, PLK2, PLPP4, PMEPA1, PMP22, PODN, PODNL1, POSTN, PPP1R18, PRKCB, PRKCDBP, PRR16, PRRX1, PRSS23, PTAFR, PTGDS, PTGS2, PTPRCAP, RAB31, RAC2, RARRES2, RASAL3, RCAN2, RCN3, RFTN1, RRAS, RUNX2, S100A4, S100B, S1PR4, SAA1, SAA2, SAMD4A, SEC14L2, SELM, SEMA7A, SERPINA3, SERPINB1, SERPINE1, SERPINF1, SERPING1, SFRP2, SFXN3, SH3KBP1, SH3TC1, SIRPA, SLC15A3, SLC1A3, SLC22A3, SLC24A3, SLC2A3, SLC38A5, SMIM3, SOD3, SPARC, SPECC1, SPI1, SPOCD1, SPON1, SPON2, SRPX2, SSPN, ST3GAL5, STAB1, STK17A, SUGCT, SULF1, SYNE1, SYNPO, SYTL2, TAGLN, TCIRG1, TFPI2, TGFB1, TGFB1I1, TGFB3, TGFBI, TGFBR2, TGM2, THBS1, THBS2, THEMIS2, THY1, TIMP1, TIMP2, TIMP3, TINAGL1, TMEM119, TMEM173, TMEM176A, TMEM176B, TMEM204, TMEM45A, TNFAIP3, TNFAIP8L2, TNFRSF12A, TNFRSF1B, TNFRSF4, TNFRSF6B, TPSB2, TREM1, TRIM47, TRPV2, TSHZ3, TSPAN11, TSPAN2, TSPAN4, TYMP, UBA7, UPP1, VASN, VCAM1, VCAN, VDR, VIM, WAS, WIPF1, WISP1, WNT2, WNT7A, ZC3H12A, ZCCHC24, ZEB2, ZFP36L2* |
| SCC5 | *A1BG, ABCA7, ACAD11, ACAP3, ADAD2, ADAMTS13, ADCY10P1, ADCY2, ADGRB1, ADM5, AGAP6, AMT, AMY2B, ANKRD23, AP1G2, APBB3, APLP1, ASIC3, ASMTL-AS1, C14orf181, C1orf220, CA11, CAMK2B, CBX7, CCDC130, CCDC154, CCDC57, CCNL1, CCNL2, CECR5-AS1, CENPT, CHKB-CPT1B, CLDN15, CLK4, CROCC, CROCCP2, CRYGS, CSAD, CUL9, CYP2D6, CYP2D7, D2HGDH, DAPL1, DCST2, DICER1-AS1, DMPK, DNAJB2, DTX1, DTX3, EGFL8, EML6, ENGASE, ENTHD2, EPHB3, FADS6, FAM156A, FAM193B, FAM73B, FAM98C, FBLL1, FBXL8, FCHSD1, GABBR1, GABRD, GIGYF1, GOLGA6L10, GOLGA8B, GPC2, GPER1, GRIK5, GUSBP11, HSF4, HSPB3, IDUA, IGSF9B, ILF3-AS1, INHA, ITGA7, KCNIP2, KIAA0895L, KIAA1683, KIFC2, L3MBTL1, LCAT, LECT1, LEKR1, LENG8, LINC00115, LINC00265, LINC00319, LINC00526, LINC00685, LINC00893, LINC00894, LINC00938, LINC00957, LINC01018, LMX1B, LOC100270804, LOC155060, LOC400927, LY6G5B, MALAT1, MAP3K10, MAPK8IP3, MIR4697HG, MROH7, MST1, MYO15A, NECTIN4, NEIL1, NFYC-AS1, NOXA1, NPDC1, NPHP3, NPIPB3, NPM2, NRBP2, NSUN5P2, OBSCN, OFD1, OR13A1, OSBP2, PAQR6, PBXIP1, PCDHB4, PCDHGA7, PCP2, PCSK4, PDXDC2P, PDZD4, PGAM2, PHKA2, PILRB, PKD1P1, PLCH2, PNPLA7, PPP1R3E, PRB3, PRCD, PRORSD1P, RAB24, RASA4CP, REM2, RGS11, RGS14, RGS9, RIC3, RIMS3, RSRP1, SDHAP1, SEC31B, SFI1, SH3GL3, SHC2, SLC25A27, SLC4A3, SNAP91, SNCAIP, SNHG10, SNHG7, SPAG17, SPEG, SPERT, SPPL2B, SRRM3, SSBP2, STMN2, STRC, TAF1C, TCEA3, TCTE3, THUMPD3-AS1, TMEM184A, TMEM74B, TNFRSF25, TNK2, TRAF5, TRIM52, TRPV1, TTC14, TTC21A, TTC32, TTLL3, TUBGCP6, URAHP, VAMP1, VCX3A, WASH3P, WASH7P, WNK4, ZDHHC8P1, ZMAT1, ZNF300, ZNF436-AS1, ZNF506, ZNF514, ZNF529, ZNF607, ZNF692, ZNF750, ZNF767P* | *ABCE1, ACSL4, ALDH1B1, BAG2, CALU, CARNMT1, CD274, CHCHD3, CTNNAL1, CTSC, FADS1, GLA, GTF2H3, IARS, KRT18, LDHA, NAA15, NAMPT, NCOA7, NDC1, NLN, NOCT, NUDCD1, PMAIP1, PSMD14, PTP4A1, TNFRSF10A, TNPO1, TUBA1C, UGCG, WARS* |
