# Supplementary material for: Transcriptional profiling of molecular pathways allows for the definition of robust lung squamous cell carcinoma molecular subtypes with specific vulnerabilities
Source: Clin Transl Med. 2023 Sep 21;13(9):e1413. doi: 10.1002/ctm2.1413 (PMC10514261; doi:10.1002/ctm2.1413)
Supplement: Supplementary file 3 — Supporting Information [file CTM2-13-e1413-s003.docx]

**SUPPORTING INFORMATION**

**METHODS**

**Data collection and processing**

SCC gene expression datasets were obtained from the Gene Expression Omnibus (GEO) and Array Express public repositories.^1,2^ Filters were applied to keep only human samples, to exclude datasets with less than 10 samples and platforms that did not cover a significant part of the transcriptome (**Figure S1)**. As a result, 36 datasets were finally included in this study constituting a total of 2,088 SCC samples (**Table S1**).

Raw microarray gene expression data were downloaded when available and subsequently processed according to the recommended method for each platform (i.e., Affymetrix, Agilent, Illumina). For TCGA-LUSC RNA-Seq dataset, gene expression quantification was downloaded from TCGA2BED FTP repository in transcripts per million (TPM).^3^

**SCC consensus pathway-transcriptional subtypes definition**

*Profiling of 50 hallmark pathways in the SCC datasets*

The activity level of 50 signaling pathways, included in the Hallmarks collection of MSigDB, was determined for all collected gene expression profiles using Gene Set Variation Analysis (GSVA) method.^4,5^ The Hallmarks collection was created by Liberzon et al. using a combination of bioinformatics and expert manual curation approaches to summarize the more than 10,000 metabolic and cancer-related pathways initially included in this database, to minimize redundancy and heterogeneity and improve interpretation.^5^ For each sample and pathway, we obtained a score between [-1, +1], with extreme values close to +1 or -1 indicating a relative strong or weak activity, respectively, of a pathway in a specific sample compared to the other samples in the dataset. GSVA score is a relative measure that depends on the number and the nature of the accompanying samples in the same dataset. Therefore, we performed a k-fold approach across 100 iterations to reduce this potential bias. In each iteration, we randomly split each dataset in five subsets of the same size (k = 5-fold) and calculated the GSVA score in one of the subsets (1/5th of the samples) and in the rest separately (4/5th of the samples). This was repeated five times, until each of the five subsets was used independently once. Thus, we obtained five different GSVA scores for each sample and pathway in each repetition. All iterations started with a different sample permutation order. As a result, we obtained 500 GSVA scores matrices (5-fold x 100 iterations).

*Consensus pathway-transcriptional subtypes identification*

Uniform Manifold Approximation and Projection (UMAP) algorithm^6^ and walktrap graph-based clustering method with Euclidean distance were used on each of the 500 GSVA scores matrices for lung SCC subpopulations identification. After obtaining these 500 potential classifications with different subpopulations of SCC samples, we conducted the following series of steps: 1) average GSVA scores for each evaluated pathway were calculated for the subgroups defined within each of the 500 iterations. We called these summary metrics *centroids*; 2) UMAP, walktrap method with Euclidean distance were applied to these centroids and consensus subtypes were identified; 3) Samples were allocated to the subtype to which they had been assigned the majority of times across the different iterations. If a tie existed between two consensus subtypes, the sample was eliminated from subsequent analyses.

**Consensus transcriptional subtypes characterization**

*Clinicopathological covariates and overall survival*

Association with clinicopathological variables (age, sex, stage and smoking status) was done using *compareGroups* package for R (V.4.2.0).^7^

Cox proportional-hazard model adjusted for age, sex, stage, smoking status, and study were used to test for the impact of our classification on overall survival (OS) rate.

*Comparison with Wilkerson et al.’s previous mRNA-based SCC classification*

The Wilkerson et al. subtype predictor centroids were used to make subtype predictions for the discovery dataset following a nearest centroid procedure as previously described.^8^

*Genomic alterations*

TCGA-LUSC dataset^9^ had somatic variant data available to evaluate the mutational burden. The total number of variants per sample was calculated excluding synonymous mutations. Using somatic single nucleotide variants (SNV), mutational signatures were inferred using the R package *SigProfilerExtractorR*.^10^ Copy number alteration (CNA) load was also evaluated in the TCGA-LUSC dataset.^9^ Genome instability was evaluated using previously calculated scores in the TCGA-LUSC dataset.^11^

*Assessment of immune cells infiltration and immune checkpoints expression*

The immune infiltrate composition of 21 cell types was deconvoluted from the gene expression profiles using GSVA.^4^ Gene signatures of the immune fractions were obtained from a previous study.^12^ Since GSVA can only deconvolute multigene signatures, we replaced two single-gene signatures (plasmacytoid dendritic cells and regulatory T cells) with their corresponding multigene signatures from another study.^13^ Additionally, when more specific categories were available for a cellular fraction (i.e., Macrophages M1 and Macrophages M2), general categories (i.e., Macrophages) were excluded. For each immune cell population, we classified samples in two categories, high infiltration, and low infiltration, using the median GSVA score as the cut-off point. Then, the percentage of samples with high infiltration of each immune cell was calculated.

To assess immune checkpoint inhibitors (ICI), activators (ICA) and T cell exhaustion status, multiple gene sets were obtained from previous studies, including lists of ICI, ICA ^14^ and T cell markers.^15^ For each marker, we divided the samples of each dataset in two categories, high expression, and low expression, using the median value as the cut-off point. Then the percentage of samples with high expression of each marker was calculated.

**Consensus transcriptional subtypes independent validation**

Subtype status of lung SCC samples in the CPTAC-3 validation cohort ^16^ was predicted using the *predict* function of the umap R package version 0.2.7.0 and a k-nearest-neighbors approximation.^6^ Briefly, for each new sample we obtained GSVA scores corresponding to the relative activity levels of the same 50 molecular pathways used to establish the consensus classification of lung SCC tumors. This step was performed following the same previously described 5-fold approach across 100 iterations. Then, the *predict* function was used on the computed GSVA scores in each iteration to map the new samples on the consensus map of lung SCC tumors. Finally, new samples subtype assignation was decided based on the most frequent label of the closest neighbors of the original classification in each iteration. Thus, after 100 iterations, each new sample had 100 potential subtype assignations. Samples were allocated to the subtype to which they had been assigned the majority of times across all the classification process.

**Assessment of potential response to therapy**

Data from three different pharmacologic datasets, GDSC, CTRPv2 and PRISM, were integrated to identify specific treatment options using *PharmacoGx* Bioconductor (RRID:SCR_006442) package.^17^ First, lung SCC cancer cell lines (SCC-CCL) were classified based on the primary tumor’s classification using the *predict* function within umap R package as previously described for the independent validation. Subtype assignation along with genomic information available from these cell lines can be found in **Figure S11**. Area above the curve (AAC) sensitivity measures for each drug and cell line were used to identify potential therapeutic vulnerabilities for the different subtypes. Importantly, *PharmacoGx* AAC values were normalized by the concentration range of the experiment in each study and take values between [0, 1]. Thus, the greater the AAC the more effective is a drug against a specific cell line. Subtypes were considered as potentially sensitive to the treatment if the average AAC value for the cell lines classified within a certain group was greater than 0.5 for at least 2 out of the 3 pharmacogenomics studies. Also, average AACs were only calculated if the treatment had been tested in at least 2 different cell lines within a subtype and study.

Transcriptional signatures of platinum therapy resistance were used to compute GSVA scores for each sample.^18^ The tested signatures were cisplatin resistance and carboplatin resistance. Kruskal Wallis test was used to test for significant differences between the subtypes.

**Subtype-specific gene expression signatures derivation and evaluation**

TCGA-LUSC dataset was used to perform differential gene expression analysis between the five SCC subtypes. TCGA-LUSC dataset was used as a representation of the discovery set because it is the biggest dataset and gene expression was measured through RNA-Seq technology, which accounts for more genes than the older microarray datasets. Two gene expression signatures were identified for each subtype, one accounting for upregulated and the other one accounting for downregulated genes when compared to the other subtypes. Genes were considered significantly up or downregulated if FDR was less than 0.05 and a difference in gene expression greater than the mean difference for all genes for at least 3/4 pairwise comparisons. These signatures were then evaluated in the CPTAC-3 lung SCC validation cohort using GSVA to see if they correlated with the corresponding subtype. For each sample, the final GSVA score was calculated as the difference between the up-signature score and the down-signature score, divided by two to maintain the score in the [-1, 1] scale.

**Data availability**

Gene expression data for SCC consensus classification were obtained from GEO and ArrayExpress public repositories. Specific information and identifiers from each dataset are available in **Table S1.**

CCLE and GDSC lung SCC cancer cell lines molecular data was obtained from <https://depmap.org> and <https://www.cancerrxgene.org/gdsc1000/GDSC1000_WebResources/Home.html>, respectively. Specific lung SCC-CCL used for this study:

| **Cell line** | **Source** |
| --- | --- |
| NCI-H2066 (RRID:CVCL_1520) | CCLE, GDSC |
| NCI-H2286 (RRID:CVCL_1545) | CCLE, GDSC |
| NCI-H520 (RRID:CVCL_1566) | CCLE, GDSC |
| LUDLU-1 (RRID:CVCL_2582) | CCLE |
| RERF-LC-A1 (RRID:CVCL_4402) | CCLE |
| HCC95 (RRID:CVCL_5137) | CCLE |
| HCC2814 (RRID:CVCL_V586) | CCLE |
| SK-MES-1 (RRID:CVCL_0630) | CCLE, GDSC |
| KNS-62 (RRID:CVCL_1335) | CCLE, GDSC |
| RERF-LC-Sq1 (RRID:CVCL_1656) | CCLE, GDSC |
| SW900 (RRID:CVCL_1731) | CCLE, GDSC |
| HLF-a (RRID:CVCL_2255) | CCLE |
| Calu-1 (RRID:CVCL_0608) | CCLE |
| NCI-H1869 (RRID:CVCL_1500) | CCLE, GDSC |
| HCC15 (RRID:CVCL_2057) | CCLE, GDSC |
| LOU-NH91 (RRID:CVCL_2104) | CCLE, GDSC |
| Sq-1 (RRID:CVCL_4900) | CCLE |
| LK-2 (RRID:CVCL_1377) | CCLE, GDSC |
| NCI-H1703 (RRID:CVCL_1490) | CCLE, GDSC |
| NCI-H2170 (RRID:CVCL_1535) | CCLE, GDSC |
| EBC-1 (RRID:CVCL_2891) | CCLE, GDSC |
| LC-1F (RRID:CVCL_1372) | CCLE, GDSC |
| NCI-H1385 (RRID:CVCL_1466) | CCLE |
| HCC1588 (RRID:CVCL_A351) | CCLE |
| COR-L32 (RRID:CVCL_2413) | GDSC |
| HARA [Human squamous cell lung carcinoma] (RRID:CVCL_2914) | GDSC |
| LC-1/sq (RRID:CVCL_3008) | GDSC |

GDSC, CTRPv2 and PRISM studies drug sensitivity data was available within the *PharmacoGx* R package.^17^

Lung SCC CPTAC-3 study gene expression and copy number alterations data was downloaded from the supplementary material of ^16^.

**REFERENCES**

1. Home - GEO - NCBI. Accessed April 16, 2023. https://www.ncbi.nlm.nih.gov/geo/

2. BioStudies. BioStudies < The European Bioinformatics Institute < EMBL-EBI. Accessed April 16, 2023. https://www.ebi.ac.uk/biostudies/arrayexpress/studies

3. Cumbo F, Fiscon G, Ceri S, Masseroli M, Weitschek E. TCGA2BED: extracting, extending, integrating, and querying The Cancer Genome Atlas. *BMC Bioinformatics*. 2017;18(1):6. doi:10.1186/s12859-016-1419-5

4. Hänzelmann S, Castelo R, Guinney J. GSVA: gene set variation analysis for microarray and RNA-Seq data. *BMC Bioinformatics*. 2013;14(1):7. doi:10.1186/1471-2105-14-7

5. Liberzon A, Birger C, Thorvaldsdóttir H, Ghandi M, Mesirov JP, Tamayo P. The Molecular Signatures Database Hallmark Gene Set Collection. *Cell Syst*. 2015;1(6):417-425. doi:10.1016/j.cels.2015.12.004

6. Konopka T. umap: Uniform Manifold Approximation and Projection. Published online February 1, 2023. Accessed April 16, 2023. https://CRAN.R-project.org/package=umap

7. Subirana I, Salvador J. compareGroups: Descriptive Analysis by Groups. Published online November 18, 2022. Accessed April 16, 2023. https://CRAN.R-project.org/package=compareGroups

8. Wilkerson MD, Yin X, Hoadley KA, et al. Lung Squamous Cell Carcinoma mRNA Expression Subtypes Are Reproducible, Clinically Important, and Correspond to Normal Cell Types. *Clin Cancer Res*. 2010;16(19):4864-4875. doi:10.1158/1078-0432.CCR-10-0199

9. Hammerman PS, Lawrence MS, Voet D, et al. Comprehensive genomic characterization of squamous cell lung cancers. *Nature*. 2012;489(7417):519-525. doi:10.1038/nature11404

10. SigProfilerExtractorR. Published online January 12, 2023. Accessed April 16, 2023. https://github.com/AlexandrovLab/SigProfilerExtractorR

11. Marquard AM, Eklund AC, Joshi T, et al. Pan-cancer analysis of genomic scar signatures associated with homologous recombination deficiency suggests novel indications for existing cancer drugs. *Biomark Res*. 2015;3:9. doi:10.1186/s40364-015-0033-4

12. Bindea G, Mlecnik B, Tosolini M, et al. Spatiotemporal Dynamics of Intratumoral Immune Cells Reveal the Immune Landscape in Human Cancer. *Immunity*. 2013;39(4):782-795. doi:10.1016/j.immuni.2013.10.003

13. Charoentong P, Finotello F, Angelova M, et al. Pan-cancer Immunogenomic Analyses Reveal Genotype-Immunophenotype Relationships and Predictors of Response to Checkpoint Blockade. *Cell Rep*. 2017;18(1):248-262. doi:10.1016/j.celrep.2016.12.019

14. Pardoll DM. The blockade of immune checkpoints in cancer immunotherapy. *Nat Rev Cancer*. 2012;12(4):252-264. doi:10.1038/nrc3239

15. Wherry EJ, Kurachi M. Molecular and cellular insights into T cell exhaustion. *Nat Rev Immunol*. 2015;15(8):486-499. doi:10.1038/nri3862

16. Satpathy S, Krug K, Beltran PMJ, et al. A proteogenomic portrait of lung squamous cell carcinoma. *Cell*. 2021;184(16):4348-4371.e40. doi:10.1016/j.cell.2021.07.016

17. Smirnov P, Safikhani Z, Eeles C, Freeman M, Haibe-Kains B. *PharmacoGx: Analysis of Large-Scale Pharmacogenomic Data*. Bioconductor version: Release (3.15); 2022. doi:10.18129/B9.bioc.PharmacoGx

18. Mucaki EJ, Zhao JZL, Lizotte DJ, Rogan PK. Predicting responses to platin chemotherapy agents with biochemically-inspired machine learning. *Signal Transduct Target Ther*. 2019;4(1):1-12. doi:10.1038/s41392-018-0034-5
